# Supplementary material for: Interactive, integrated analysis of single-cell transcriptomic and phylogenetic data with PhyloVision
Source: Cell Rep Methods. 2022 Apr 25;2(4):100200. doi: 10.1016/j.crmeth.2022.100200 (PMC9046453; doi:10.1016/j.crmeth.2022.100200)
Supplement: Document S1. Figures S1–S4 [file mmc1.pdf]

Cell Reports Methods, Volume *2*

Supplemental information

Interactive, integrated analysis of single-cell transcriptomic and phylogenetic data with PhyloVision

Matthew G. Jones, Yanay Rosen, and Nir Yosef

SUPPLEMENTARY FIGURES AND LEGENDS

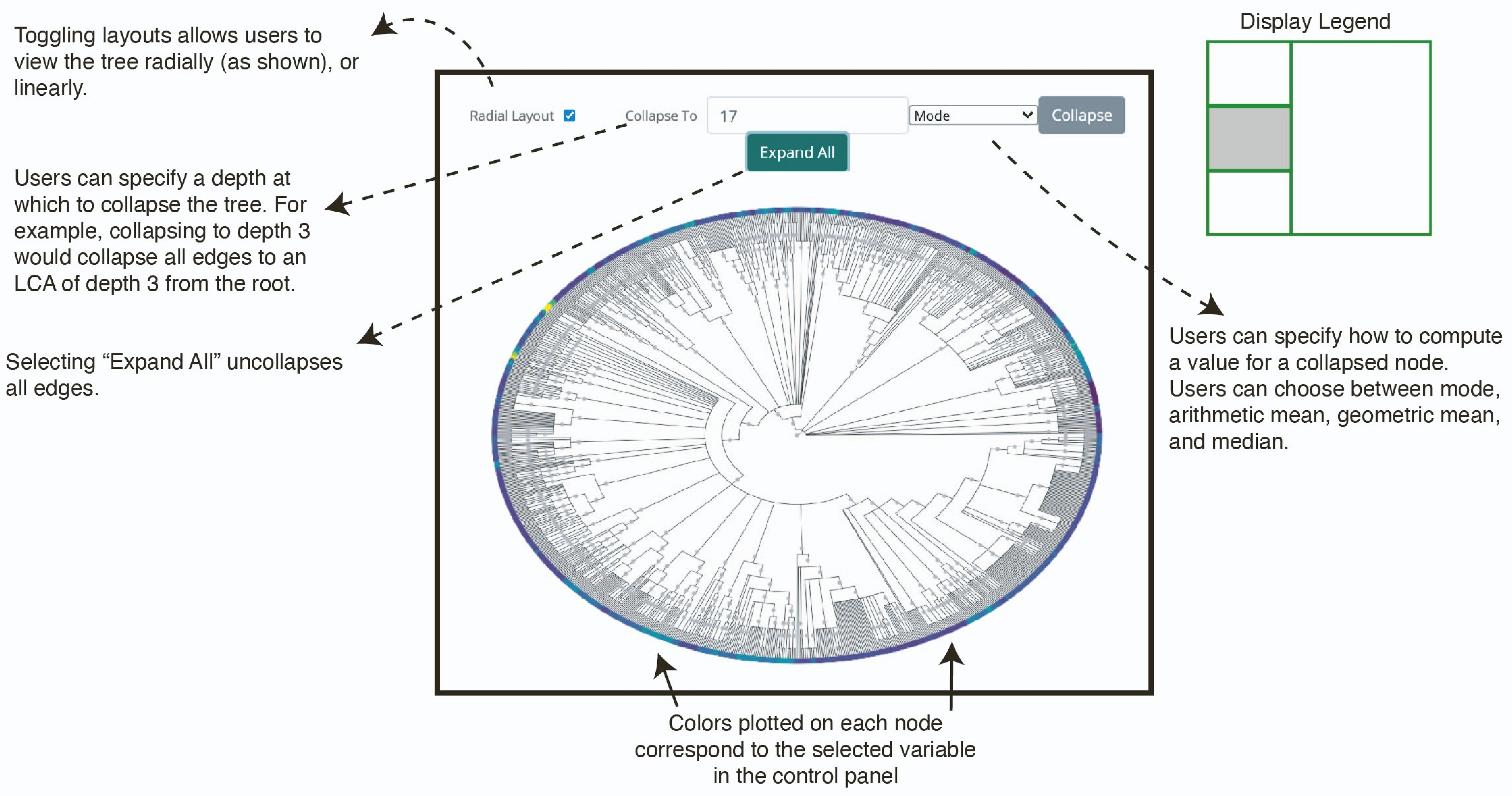

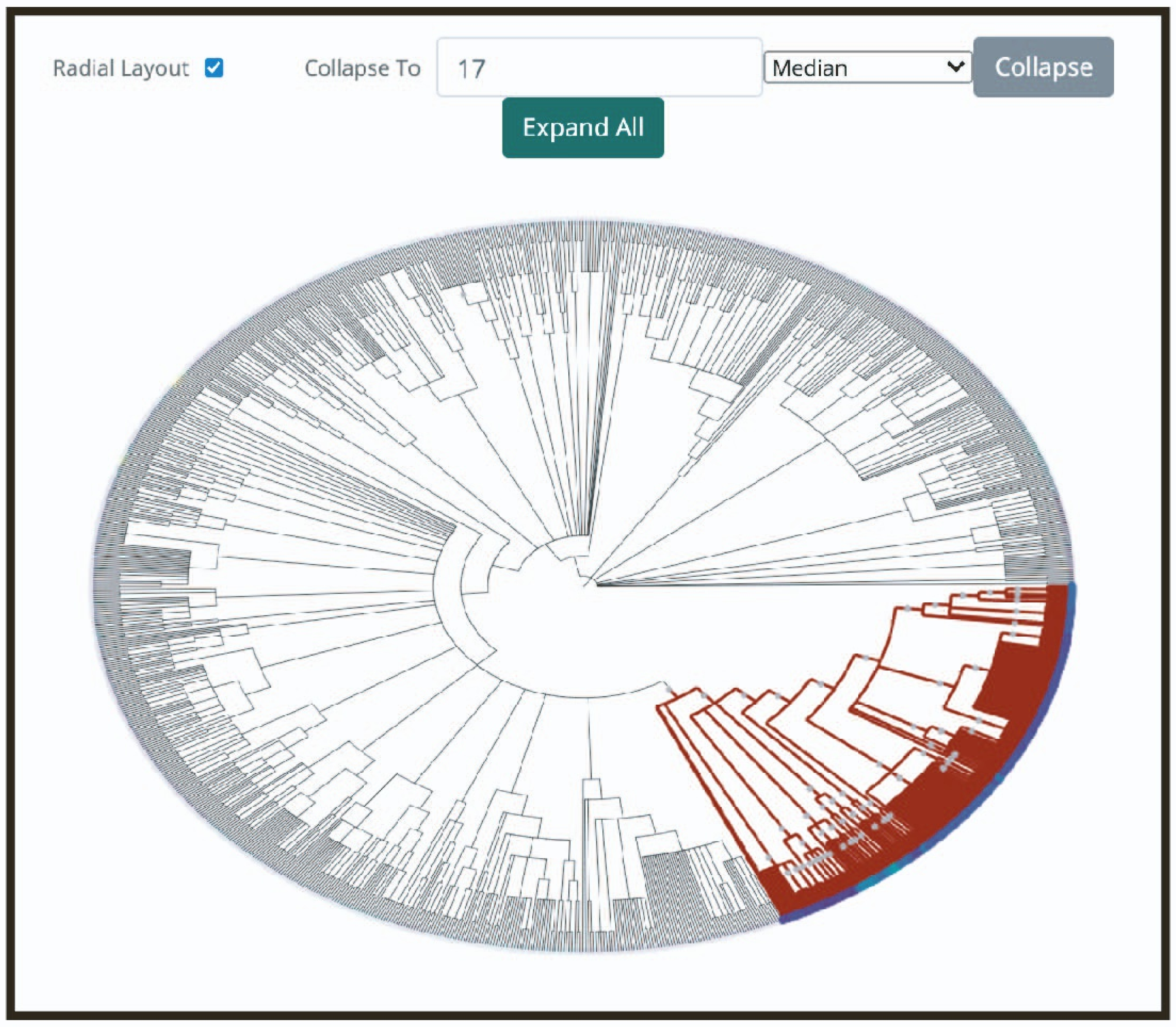

Clades can be selected by clicking with the left-mouse button on an internal node. These selections can be propagated to the cell projection viewer or the differential expression analysis tool.

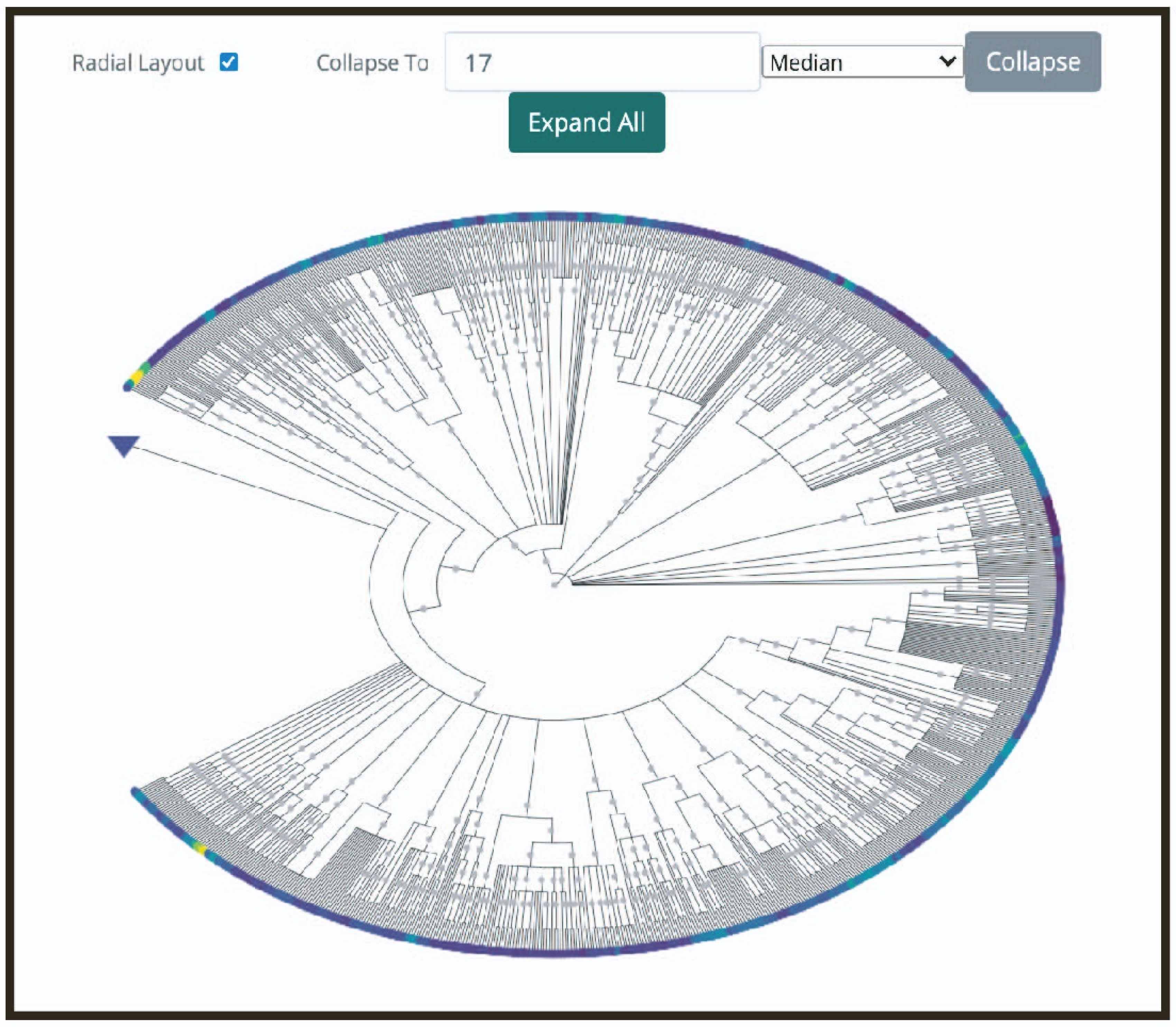

Clades can be collapsed individually by clicking with the right-mouse button on an internal node. Values displayed will be computed using the specified function (e.g., median)

Figure S1: Elements of the PhyloPlot interface, related to Figure 1. A tutorial over the features of the PhyloPlot.js interface used in the *PhyloVision* web-based report. Screenshots indicate how to interact with the object, including how to select, collapse, and expand clades.

“Hotspot” mode can be selected in the top right corner of the web-report

Display Legend

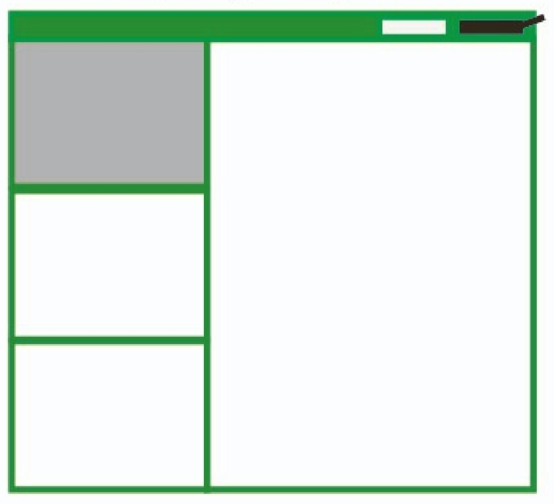

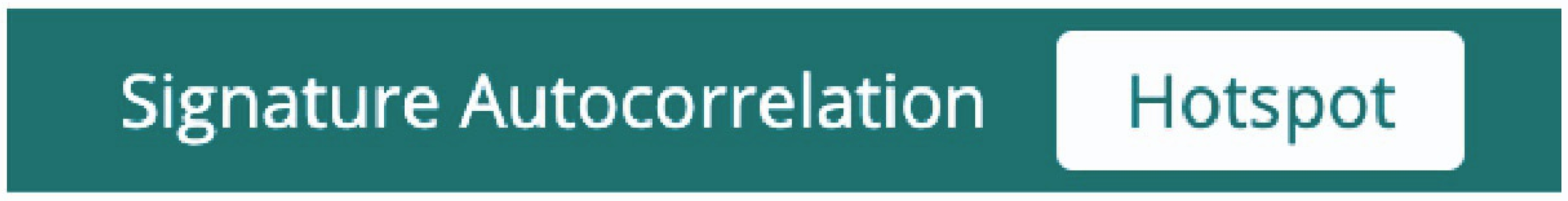

The values in the first column, labeled “Overlap” display the enrichment statistic between the genes in the Hotspot module and signature

The control panel in “Hotspot” mode contains drop down panels for each Hotspot gene module identified.

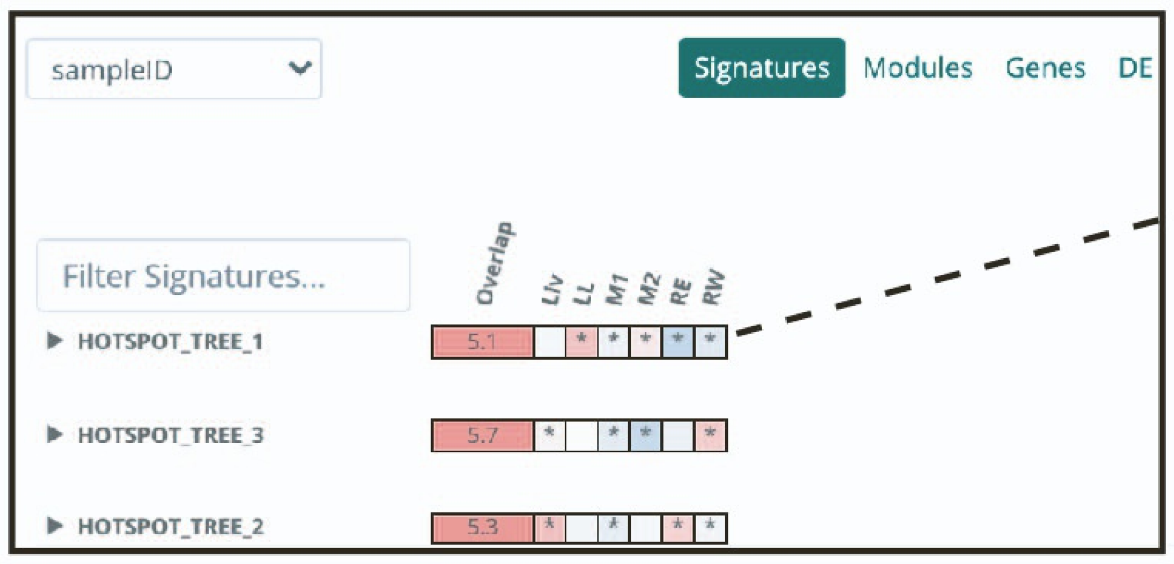

Clicking on the arrow next to a module reveals a table with entries for each signature that significantly overlaps with the module

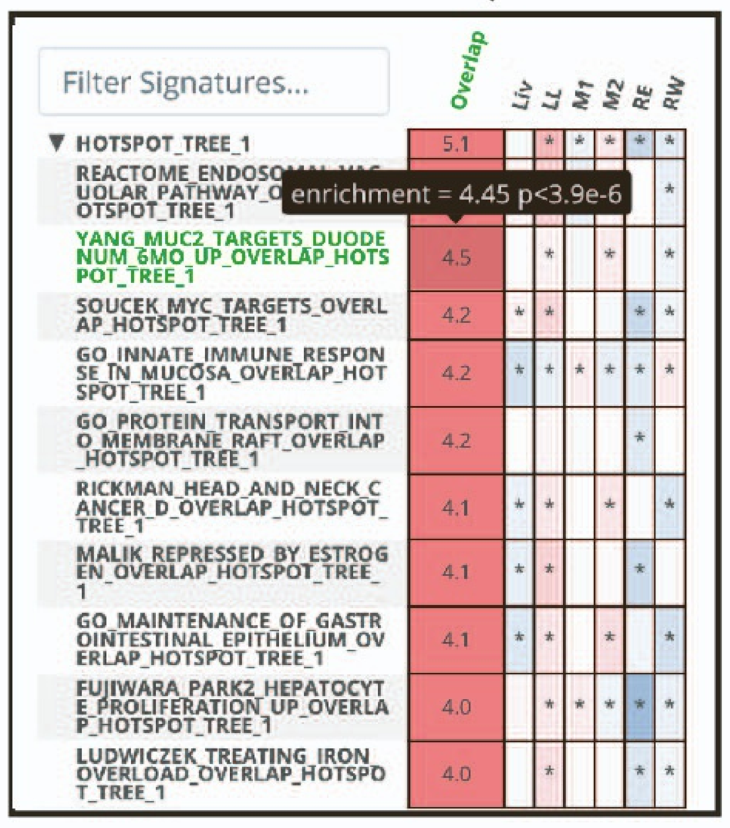

Clicking on a row displays the signature score on the cell-projection and tree. In Hotspot mode, signature scores are computed on the overlapping genes.

Hovering over a cell dispalys its enrichment statistic and FDR-corrected p-value.

Additional columns show the result of 1 vs. All differential signature tests for the selected grouping variable.

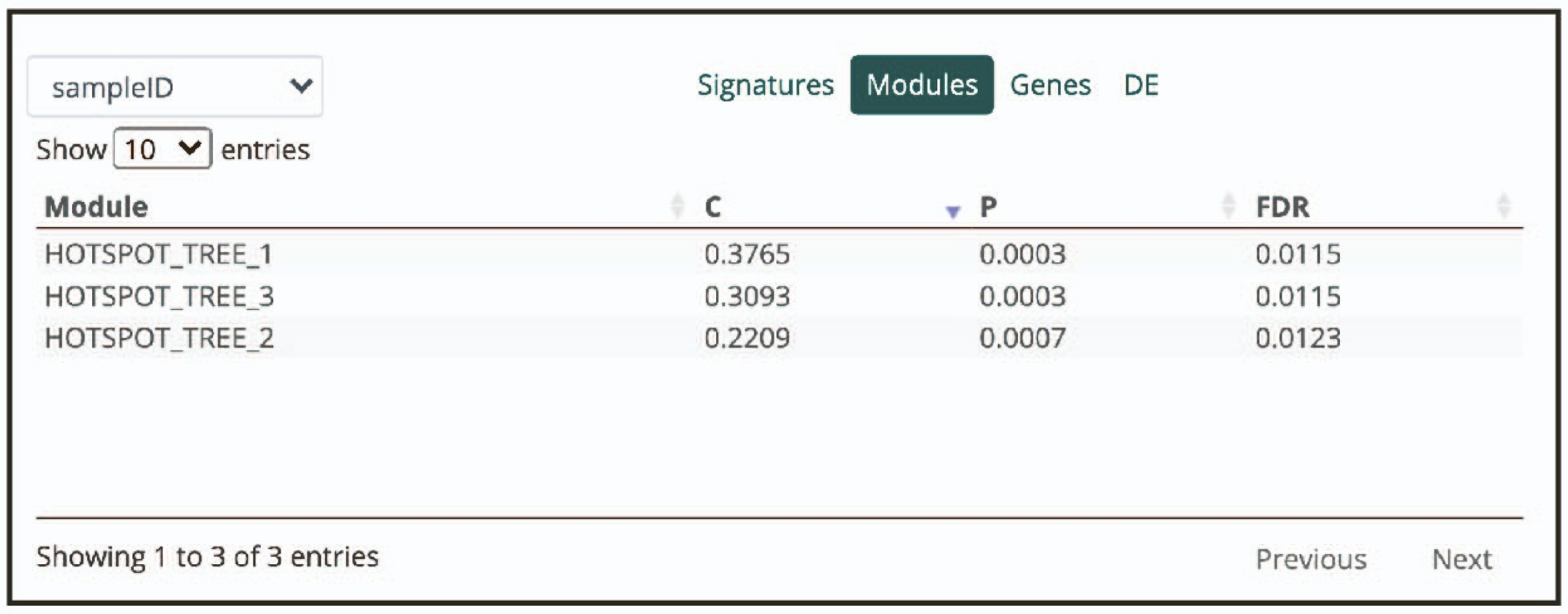

Hotspot module statistics can be viewed under the “Modules” tab.

The Geary’s C, p-value, and FDR value are displayed for each module.

The number of modules per page can be selected.

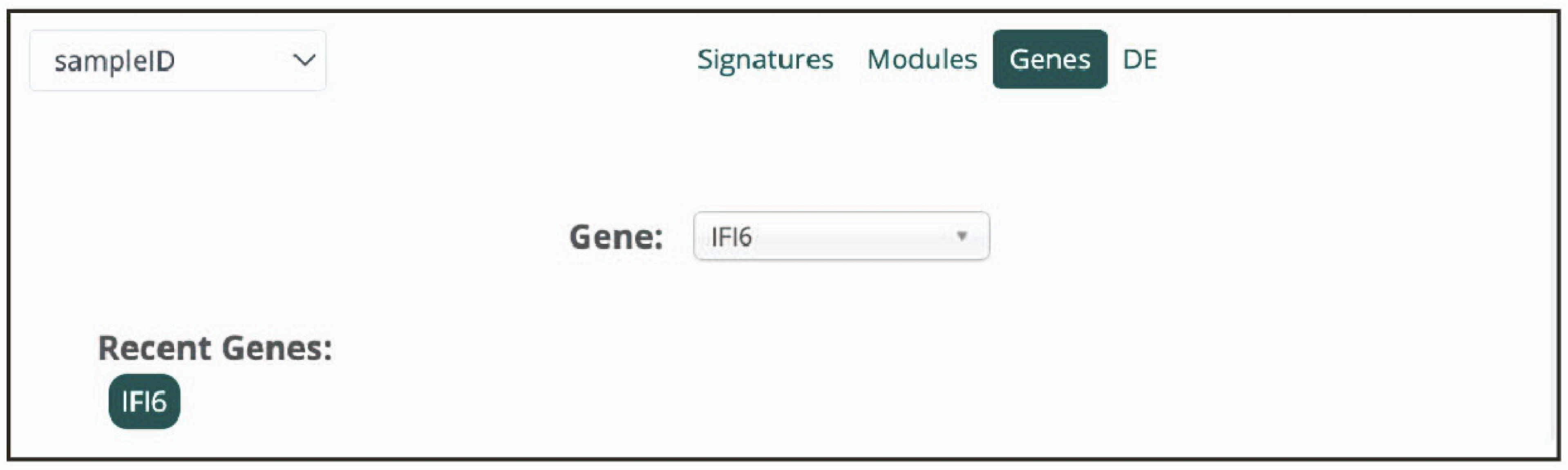

Gene expression can be overlaid onto the tree and two-dimensional embedding.

Genes can be selected via the dropdown menu.

Recently used genes can be selected under the “Recent Genes” list.

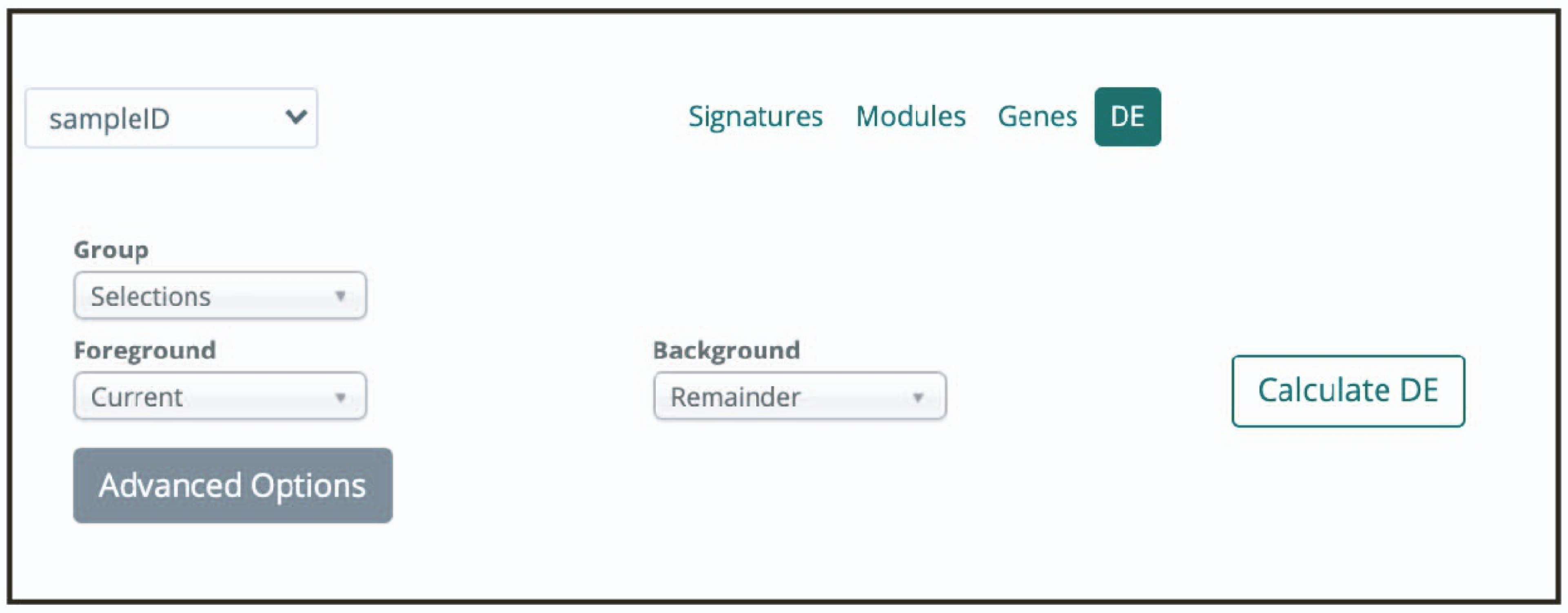

A differential expression anlaysis can be conducted under the “DE” tab. Various tests can be selected under “Advanced Options.”

A selected group can be compared against another group, or the remainder of cells if no group is specified.

Figure S2: Elements of the *Hotspot* mode control panel, related to Figure 1. Users can toggle between signature autocorrelation and *Hotspot* modes in the top-right corner of the web-based report. In *Hotspot* mode, each module is presented in a collapsable table. Elements in the table are gene signatures that significantly overlap with the *Hotspot* module (see Methods). Users can also view the *Hotspot* summary statistics with the "Modules" tab, where the Geary's C, p-values, and FDR of each module are displayed. Users can also select specific genes for displaying on the phylogeny and scatter plot, as well as perform differential expression tests. Multiple differential expression tests are supported under "Advanced Options".

Display Legend

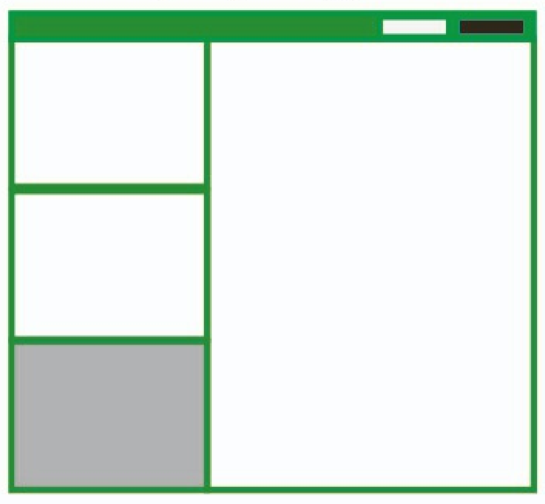

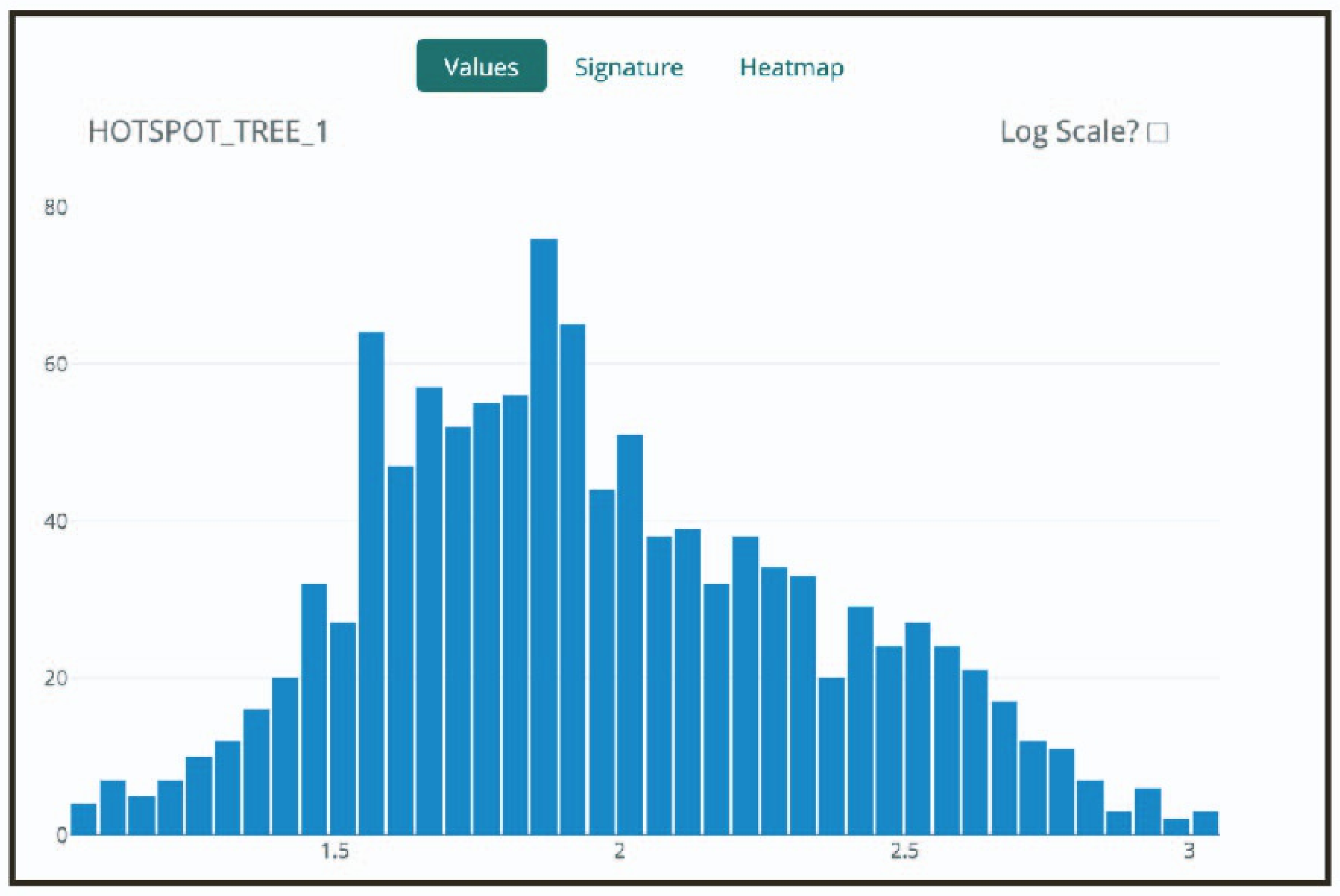

When selecting a Hotspot module, the exploration panel displays the distribution of scores.

Selecting the “log scale” radio button shows the logged distribution.

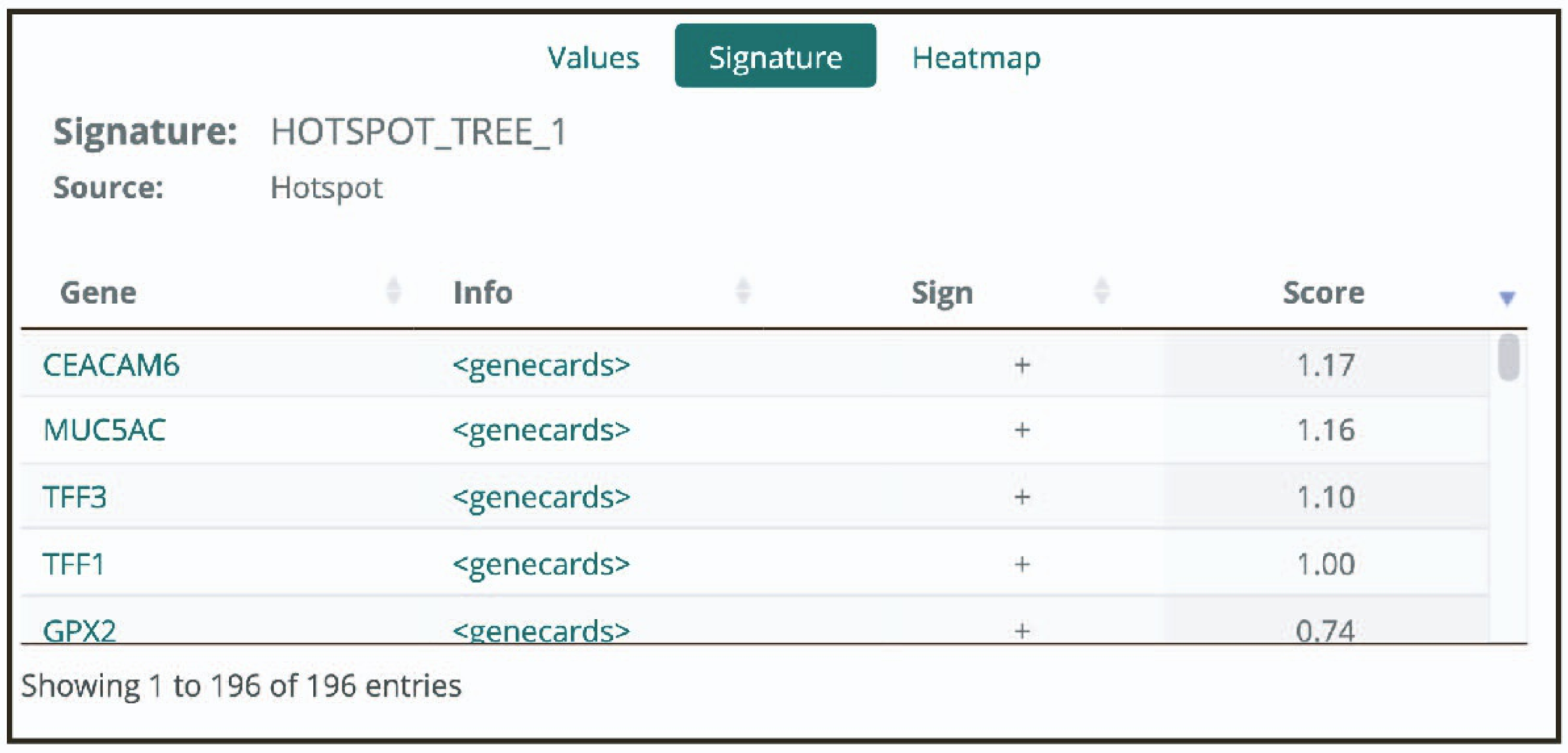

Signature: HOTSPOT_TREE_1
Source: Hotspot

Gene	Info	Sign	Score
CEACAM6	<genecards>	+	1.17
MUC5AC	<genecards>	+	1.16
TFF3	<genecards>	+	1.10
TFF1	<genecards>	+	1.00
GPX2	<genecards>	+	0.74

Showing 1 to 196 of 196 entries

The “Signature” tab shows the genes in the Hotspot gene module.

Clicking on a gene name displays the counts on the cell-projection and tree.

Clicking on “genecards” directs the user to the gene-card entry for the gene of interest

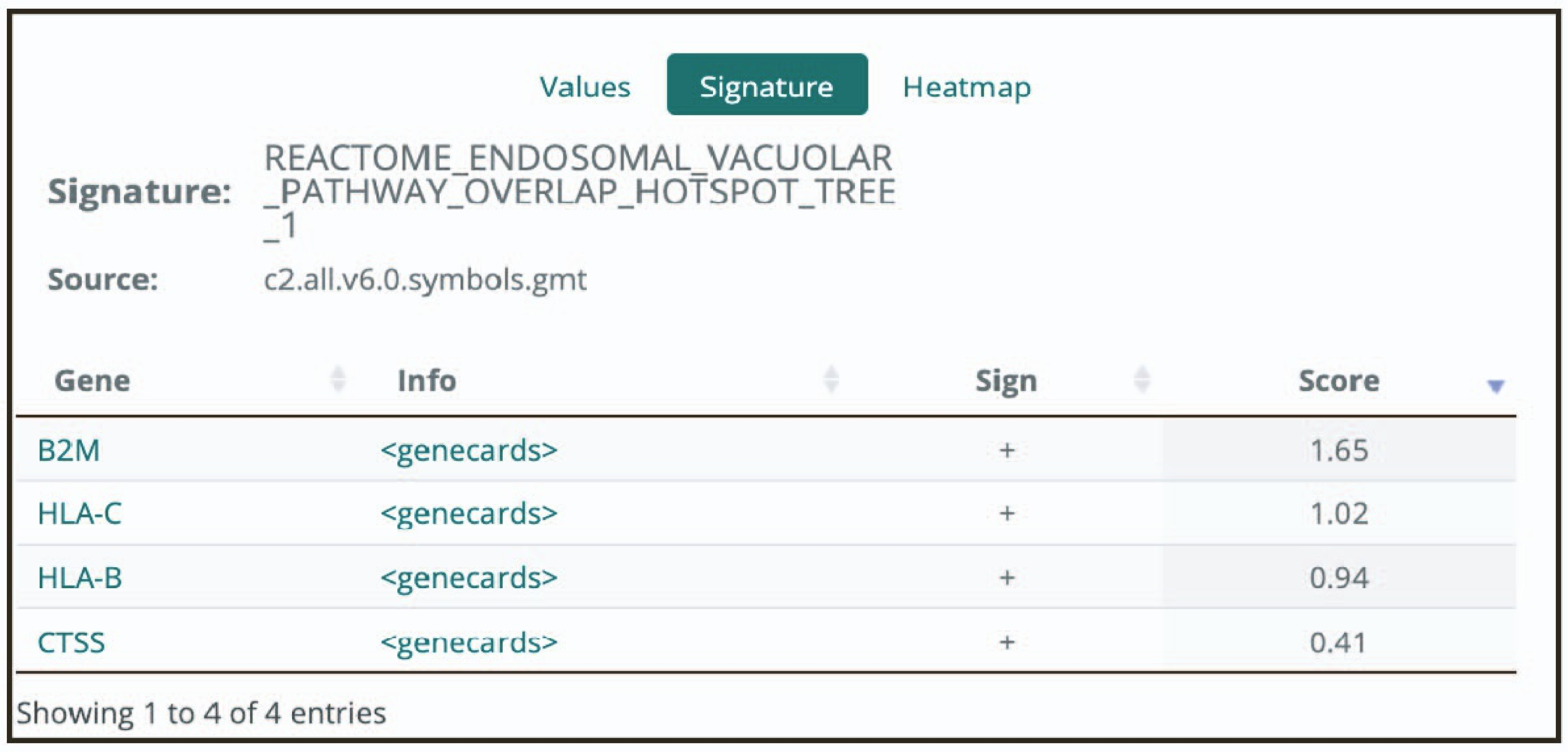

Signature: REACTOME_ENDOSOMAL_VACUOLAR_PATHWAY_OVERLAP_HOTSPOT_TREE_1
Source: c2.all.v6.0.symbols.gmt

Gene	Info	Sign	Score
B2M	<genecards>	+	1.65
HLA-C	<genecards>	+	1.02
HLA-B	<genecards>	+	0.94
CTSS	<genecards>	+	0.41

Showing 1 to 4 of 4 entries

If a signature is selected in the Hotspot mode, the overlapping genes are dispalyed in the “Signature” information tab.

Figure S3: **Elements of the *Hotspot* mode signature exploration panel, related to Figure 1.** Users can view the distribution of scores in the *Hotspot* mode on both normal and log scales. If the *Hotspot* module is selected, scores are the *VISION*-computed score for the genes in the *Hotspot* module. If a signature is selected, the distribution displayed is the signature score of only the genes overlapping between the *Hotspot* module and the gene signature. Users can also view the genes in a *Hotspot* module, or in the overlap between a gene signature and *Hotspot* module.

Display Legend

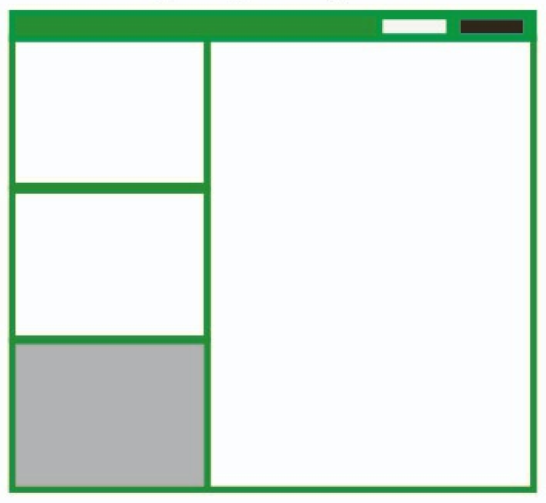

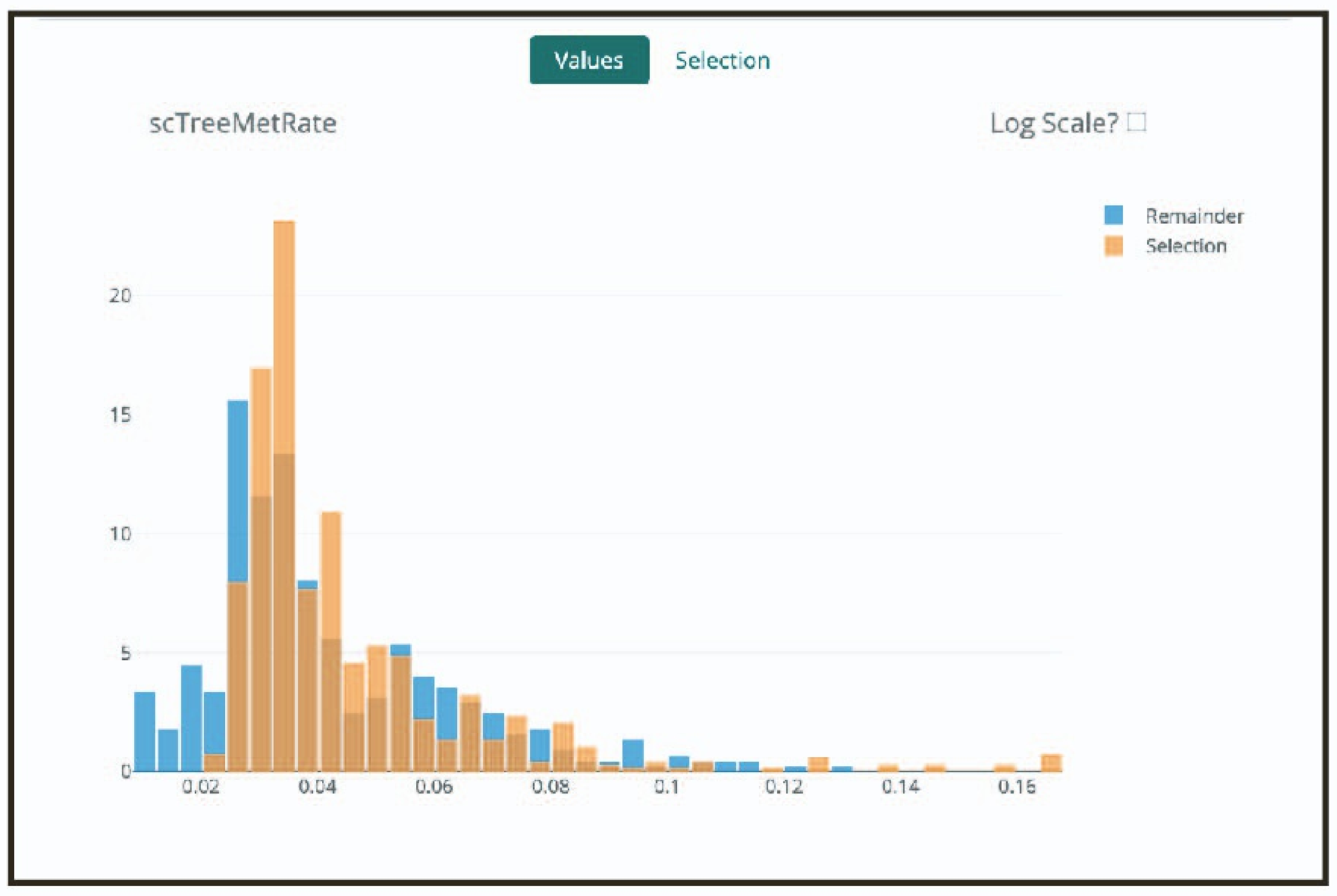

If a cell selection is active, the "Values" distribution will show two distributions: one for the selected cells (orange) and one for the rest of the cells (blue).

Values Selection

Cells Selected: 678

Numeric Variables	Min	Median	Max
LineageGroup	4	4	4
nGenes	317	1860	5655
NUM_INTBC	2	7	22
nUMI	2061	7016	52090
scTreeMetRate	0.02185	0.03641	0.1677
StaticMetScore	0.7626	0.7626	0.7626
TissueDispersion	0.7626	0.7626	0.7626
TreeMetRate	0.05845	0.05845	0.05845
TS_UMI	10	61	675

Categorical Variables	Top Value	Percent	Entropy
▶ VISION_Clusters_Tree	Cluster 6	34.2%	2.279
▶ Sample2	M	64.5%	1.231
▶ sampleID	M1	40.9%	2.023

The "Selection" tab will show a breakdown of meta data within the selected cell population.

Summary statistics are shown for numerical meta data.

The most frequent category, percentage of cells reporting this category, and entropy of the variable are shown for categorical meta data.

A list of selected calls can be downloaded with the arrow button next to "Cells Selected".

Categorical Variables	Top Value	Percent	Entropy
▼ VISION_Clusters_Tree	Cluster 6	34.2%	2.279

VISION_Clusters_Tree	
Cluster 6	34.2%
Cluster 10	30.5%
Cluster 7	11.2%
Cluster 9	8.7%
Cluster 5	8.6%
Cluster 8	6.8%

Clicking on an arrow next to a categorical variable reveals a more granular breakdown of the composition of a given selection of cells.

Figure S4: **Investigating cell selections, related to Figure 1.** Users can investigate a selection with respect to its distribution of the plotted value as compared to the background (i.e., non-selected cells). *PhyloVision*'s UI also provides a breakdown of metadata within a given selection, including summary statistics over numerical metadata as well as measures of entropy and composition of categorical metadata.
