# Supplementary material for: Interactive, integrated analysis of single-cell transcriptomic and phylogenetic data with PhyloVision
Source: Cell Rep Methods. 2022 Apr 25;2(4):100200. doi: 10.1016/j.crmeth.2022.100200 (PMC9046453; doi:10.1016/j.crmeth.2022.100200)
Supplement: Document S2. Article plus supplemental information [file mmc3.pdf]

Cell Reports
Methods

Report

Interactive, integrated analysis of single-cell transcriptomic and phylogenetic data with PhyloVision

Graphical abstract

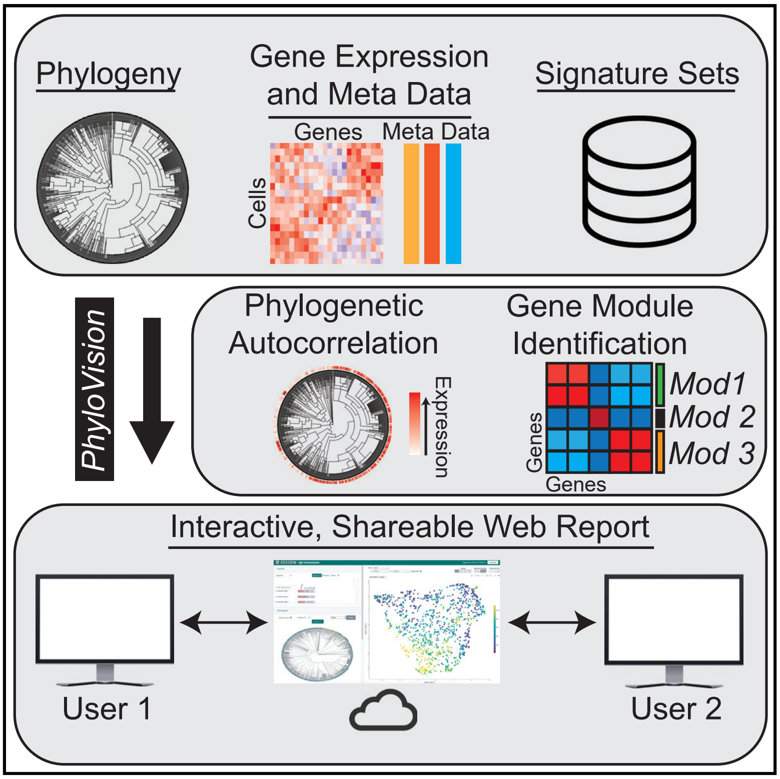

Highlights

- PhyloVision quantifies phylogenetic autocorrelation of gene expression signatures
- PhyloVision identifies *de novo* heritable gene expression modules
- PhyloVision creates a shareable, web-based report for exploring datasets
- Identification of heritable gene modules associated with metastatic behavior

Authors

Matthew G. Jones, Yanay Rosen, Nir Yosef

Correspondence

niryosef@berkeley.edu

In brief

Jones et al. present "PhyloVision," a computational resource for analyzing single-cell lineage-tracing data. PhyloVision implements an integrated framework for identifying gene expression modules associated with lineage relationships, as well as enabling collaborative projects by generating an interactive, web-based report for data exploration that can be shared among colleagues.

Jones et al., 2022, Cell Reports Methods *2*, 100200
April 25, 2022 © 2022 The Author(s).
https://doi.org/10.1016/j.crmeth.2022.100200

CellPress

Cell Reports Methods

CellPress
OPEN ACCESS

Report

Interactive, integrated analysis of single-cell transcriptomic and phylogenetic data with PhyloVision

Matthew G. Jones,[1,2,3,4,7] Yanay Rosen,[1,7] and Nir Yosef[1,2,5,6,8,*]
[1]Department of Electrical Engineering and Computer Science, University of California, Berkeley, Berkeley, CA 94720 USA
[2]Center for Computational Biology, University of California, Berkeley, Berkeley, CA 94720 USA
[3]Integrative Program in Quantitative Biology, University of California, San Francisco, San Francisco, CA 94143, USA
[4]Whitehead Institute, Cambridge, MA 02142 USA
[5]Chan Zuckerberg Biohub Investigator, San Francisco, CA 94158 USA
[6]Ragon Institute of Massachusetts General Hospital, MIT and Harvard University, Cambridge, MA 02114 USA
[7]These authors contributed equally
[8]Lead contact
*Correspondence: niryosef@berkeley.edu
https://doi.org/10.1016/j.crmeth.2022.100200

MOTIVATION Technologies enabling investigators to simultaneously profile the lineage and transcriptional states of single cells hold great promise for profiling dynamic processes such as tumor growth or organ development. Yet the analysis of these data requires the deployment of sophisticated computational tools that might preclude users without computational training from deriving insights. Here, we have developed an integrated analysis pipeline and user interface, PhyloVision, that performs fundamental analyses for these multimodal lineage-tracing assays and creates a web-based report to explore the cells' lineage relationships, transcriptomes, and association. The report can be shared online to better enable collaborative projects.

SUMMARY

Recent advances in CRISPR-Cas9 engineering and single-cell assays have enabled the simultaneous measurement of single-cell transcriptomic and phylogenetic profiles. However, there are few computational tools enabling users to integrate and derive insight from a joint analysis of these two modalities. Here, we describe "PhyloVision": an open-source software for interactively exploring data from both modalities and for identifying and interpreting heritable gene modules whose concerted expression are associated with phylogenetic relationships. PhyloVision provides a feature-rich, interactive, and shareable web-based report for investigating these modules while also supporting several other data and meta-data exploration capabilities. We demonstrate the utility of PhyloVision using a published dataset of metastatic lung adenocarcinoma cells, whose phylogeny was resolved using a CRISPR-Cas9-based lineage-tracing system. Together, we anticipate that PhyloVision and the methods it implements will be a useful resource for scalable and intuitive data exploration for any assay that simultaneously measures cell state and lineage.

INTRODUCTION

Cellular lineages underlie several important biological phenomena—from embryogenesis to differentiation to cancer progression—and understanding the nature and dynamics of these lineages remains a central focus of research. Indeed, the piecing together of the developmental lineage of *Caenorhabditis elegans* by Sulston and colleagues via visual observations (Sulston et al., 1983) has facilitated decades of critical work using the deterministic development of *C. elegans* as a model system to study development (Packer, 2019), aging (Kenyon, 2010), and even human diseases like neurodegeneration (Lu et al., 2014). Yet, many higher-order organisms cannot be studied by visual observation alone, and thus, a robust understanding of cell lineages underlying these organisms remains elusive. To this end, several technologies have emerged to track cellular lineages over varying timescales, as reviewed in previous work (Kester and van Oudenaarden, 2018; McKenna and Gagnon, 2019).

Recently, the integration of CRISPR-Cas9-based engineering and single-cell sequencing has enabled the synthetic tracing of cellular lineages at unprecedented resolution (Frieda et al., 2017; McKenna et al., 2016; Kalhor et al., 2017; Raj et al., 2018; Chan et al., 2019; Bowling et al., 2020). Several of these technologies enable the simultaneous measurement of cell

This is an open access article under the CC BY license (http://creativecommons.org/licenses/by/4.0/).

lineage and transcriptomic state via single-cell RNA sequencing (scRNA-seq), thus creating exciting opportunities to study the transcriptional evolution of dynamic processes and motivating innovative approaches for integrating these two critical modalities (Wagner and Klein, 2020). As with high-dimensional measurements like those from scRNA-seq, it is clear that specialized, interactive tools for data exploration, visualization, and analysis are necessary for realizing the full potential of these lineage-tracing assays.

There exist several useful software tools for visualization of phylogenetic or lineage-tracing data. For example, the interactive Tree of Life (iTOL; Letunic and Bork, 2021) is a scalable web-server-based tool that allows users to upload tree structures and various annotation files for interactive viewing. However, to indefinitely host and share these reports requires a paid subscription. More recently, CeLaVi was introduced as a publicly available software tool for generating interactive, web-based reports expressly for cell-lineage viewing (Salvador-Martínez et al., 2021). Although both tools are scalable to up to thousands of cells and are versatile for integrating various data modalities (e.g., gene expression measurements and spatial location) with phylogenies for visualization, they do not offer capabilities for joint analysis and automated interpretation of information on lineages and gene expression.

Here, we introduce PhyloVision: an open-source, interactive analysis and visualization tool that is expressly built for integrating single-cell gene expression and lineage data. PhyloVision builds on useful existing work, like iTOL and CeLaVi, for interactively visualizing phylogenies while possibly overlaying the expression of individual genes. In addition to this, however, PhyloVision also employs other analysis frameworks developed by our group for automated interpretation of the variation in gene expression across the lineage structure. Specifically, PhyloVision supports features that identify heritable gene expression programs and interprets these programs using gene signature enrichment analysis.

To demonstrate the utility of PhyloVision, we apply it to a clone of 1,127 cells from a recent CRISPR-Cas9 lineage-tracing dataset investigating metastatic spread in a mouse model of lung adenocarcinoma. In doing so, we show that the derived statistics and web-based user interface can be used to effectively characterize subpopulations within this aggressive tumor population. These molecular characterizations, not discussed in the original study, can be used to generate hypotheses about how metastatic ability evolves within a tumor subpopulation.

PhyloVision is distributed publicly on Github at https://github.com/YosefLab/VISION. Along with the software, we include several tutorials and example reports of published datasets allowing users to explore the user-interface. Additionally, we include a detailed manual and description of the user-interface in the supplemental information.

RESULTS

PhyloVision is an integrated pipeline for interactive analysis of single-cell expression and lineage profiles

PhyloVision is simultaneously a tool for interactive exploration of multimodal single-cell lineage-tracing data using our web-based front end and for analysis of the evolutionary dynamics of expression data. Our interactive web-based report is built on our VISION front-end (DeTomaso et al., 2019). Here, we have developed an interactive phylogeny viewer and have integrated it into the default interface, enabling the user to select cells (for visualization on a low-dimensional embedding of the respective scRNA-seq data or for differential expression analysis), perform various manipulations on the observed tree (e.g., node collapsing), and overlay gene expression, signature scores, or other data onto the leaves of the tree (Figures 1 and S1–S4; Video S1). The dynamic phylogeny viewer is scalable, allowing low-latency selections and subtree collapsing for large trees (we tested trees of up to ~4,000 leaves). Importantly, a web report generated by a user can be viewed locally, shared privately among colleagues, or staged publicly on a web server; moreover, users can download the state of any report for reproducing visualizations separately.

While the interactive web report is a useful tool for data exploration, the PhyloVision pipeline additionally supports statistical analysis for deriving joint insight from the expression and lineage data (Figure 2). In this, PhyloVision takes as input (at a minimum) an expression data matrix, a phylogeny, and a collection of gene signatures, each representing a certain pathway or a transcriptional response to a certain change in conditions (as publicly available from resources like MSigDB; Subramanian et al., 2005). In one mode of analysis, PhyloVision will conduct a phylogenetic autocorrelation analysis with the user-defined gene signatures. Here, for a given signature, a score will be computed for each cell with VISION (DeTomaso et al., 2019), indicating the cumulative activity of the respective genes (note that the score also accounts for "signed" transcriptional response signatures, in which one subset of genes is marked as up-regulated and another as down-regulated). An autocorrelation statistic will be then computed by evaluating the consistency of the signature scores between nearby cells on the phylogeny, using Geary's C statistic (Geary, 1954) as in DeTomaso et al., 2019 (STAR Methods). With this analysis, a user can identify gene signatures that are significantly associated with the tree structure, suggesting evolutionary patterns of interest. Our PhyloVision pipeline also includes modules to analyze user-provided meta-data, (e.g., the tissue of origin, extent of somatic mutations, or cell-level fitnesses inferred with external models; Neher et al., 2014), quantify cell-level plasticities with respect to categorical data (Yang et al., 2021), and interactively visualize these cell-level data (STAR Methods). Together, these "signature-level" analyses enable users to identify cell-level properties whose variation is consistent with the tree structure and to highlight phenotypes that represent subclonal, heritable phenotypes.

In another mode of analysis, users can identify *de novo* gene modules (i.e., not guided by pre-defined signatures) that are learned from the intersection of phylogeny and expression data using our Hotspot algorithm (DeTomaso and Yosef, 2021). Briefly, this analysis uses autocorrelation to identify individual genes whose expression is consistent with the phylogeny—namely, genes that are expressed at a more similar level in phylogenetically adjacent cells than in cells that are distant. It then uses a pairwise extension of the autocorrelation

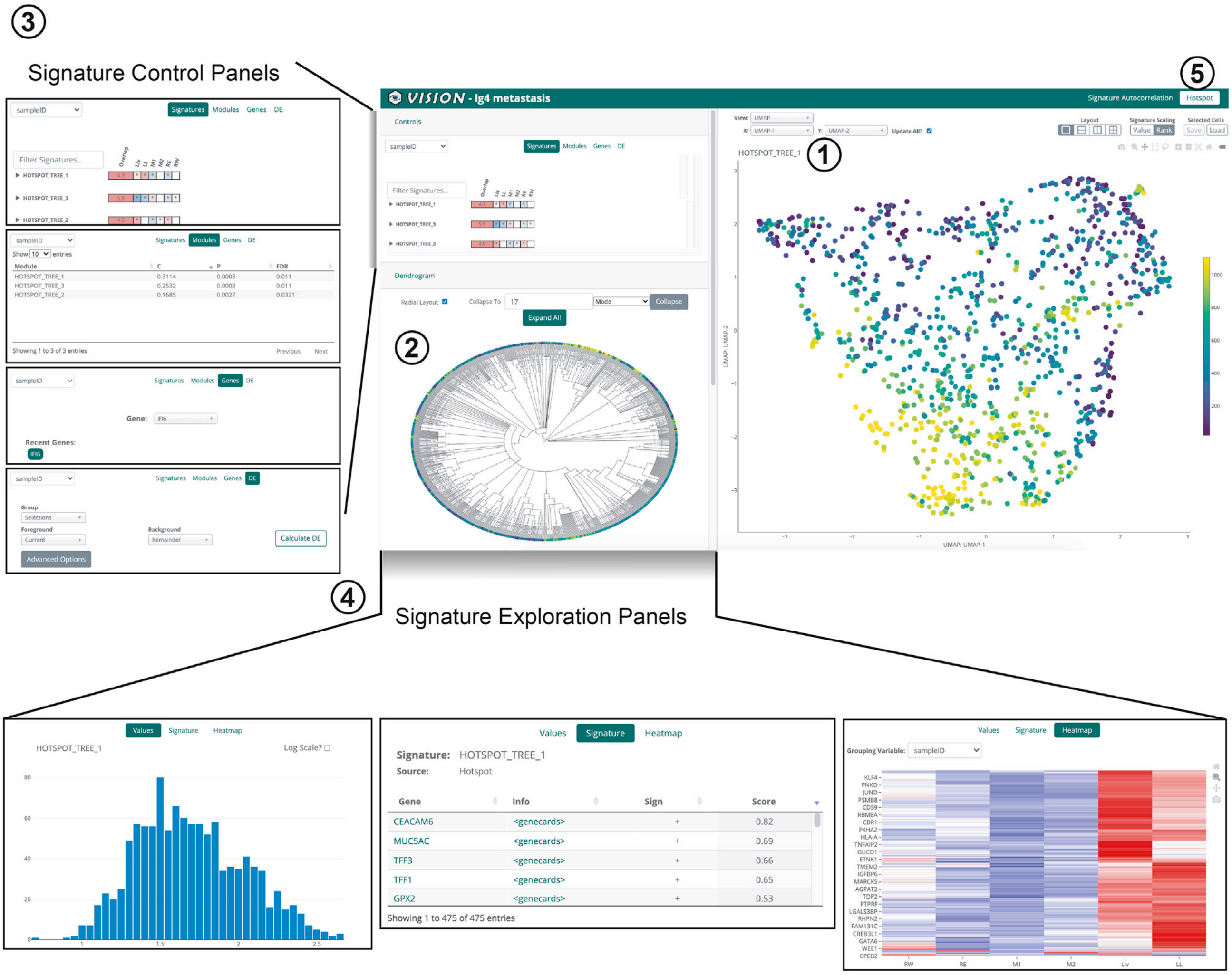

Figure 1. Overview of the PhyloVision interactive UI

PhyloVision's user interface (UI) is a web-based, feature-rich report that can be hosted locally or externally. PhyloVision incorporates four main panels into viewing. First is a panel for visualization of two-dimensional single-cell RNA-seq projections (e.g., a UMAP projection) or, alternatively, coordinates (e.g., from spatial transcriptomics datasets; inset 1). Second is a panel for interactive visualization of a phylogeny relating all cells (inset 2) that enables selection, collapsing, and variable layouts (radial or linear). Third is a control panel for selecting values to be overlaid onto the phylogeny and two-dimensional visualization panel, evaluating statistics associated with each signature or module, plotting a gene's expression, or performing differential expression analysis, with each signature or module, plotting a gene's expression, or performing differential expression analysis. In the default "Signature Autocorrelation" mode, signatures are clustered using a Gaussian mixture model to group together signatures with similar distributions (STAR Methods; inset 3). Note that here the Hotspot mode is shown, which operates "bottom up": first finding heritable gene modules and then analyzing their over-representation (enrichment) in user-provided signatures. The alternative mode, "Signature Autocorrelation," that operates directly on the user signatures is described in DeTomaso et al., 2019. Users can control the analysis mode by toggling between "Signature Autocorrelation" and "Hotspot" (inset 5). Fourth is an exploration panel for inspecting the value distribution, gene membership, and expression heatmaps of each user-provided gene signature or automatically identified Hotspot module (inset 4). See also Figures S1–S4 and Video S1.

statistic to arrange these genes into modules whose expression patterns on the phylogeny are similar, thus representing conserved transcriptomic modules that each operate in a concerted fashion. The PhyloVision pipeline adds interpretability to the Hotspot modules by assessing the overlap between their respective gene sets and the user-provided gene signatures. PhyloVision provides a quantification of this overlap with an enrichment statistic and an assessment of statistical significance. Together, these two analyses enable a user to identify important sources of transcriptomic variation on the phylogeny, as well as discover new and interpretable gene sets.

Case study: Analysis of a metastatic lung adenocarcinoma tumor with PhyloVision

To demonstrate PhyloVision's usefulness in interrogating data from multimodal single-cell lineage-tracing technologies, we applied the pipeline to a clone from our recently published dataset in which we studied the metastatic behavior of an aggressive

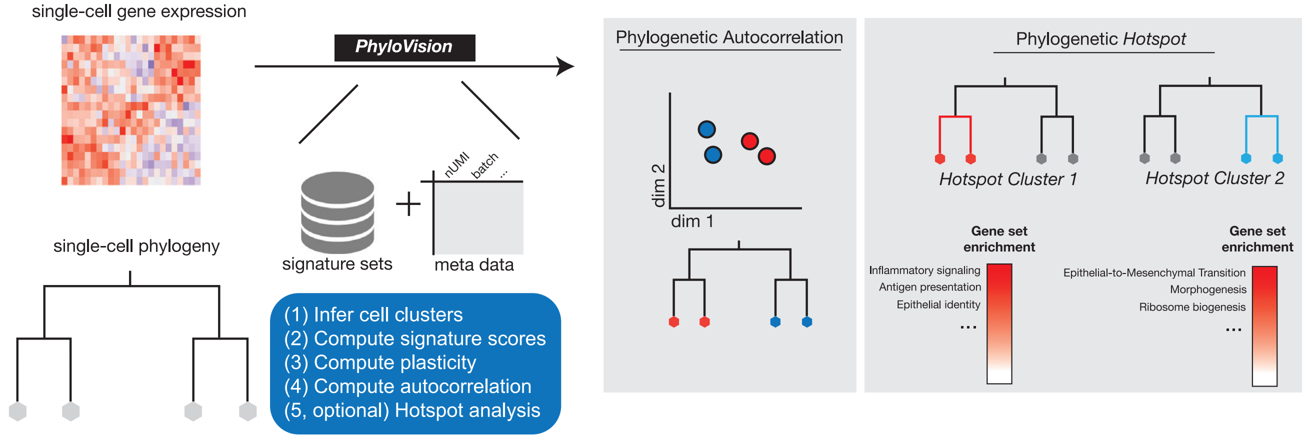

Figure 2. The PhyloVision analysis pipeline

A simplified schematic representation of the PhyloVision pipeline. PhyloVision takes as input a gene expression matrix, a phylogeny, gene signature sets, and optionally meta-data associated with each cell. Signature scores are computed for each cell in the dataset and evaluated with phylogenetic autocorrelation (STAR Methods). Additionally, plasticity indices are computed for each categorical meta-data (STAR Methods). Upon user specification, PhyloVision performs Hotspot gene module identification using the phylogeny as a latent space. Modules can be interpreted by assessing the enrichment score between signature gene sets and module gene sets.

human lung adenocarcinoma cell line in a xenograft mouse model (Quinn et al., 2021).

As previously described, we used our CRISPR-Cas9 lineage-tracing technology (Chan et al., 2019) to trace approximately 100 clones over the course of 2.5 months as each clone metastasized between tissues in a mouse model of lung adenocarcinoma. In this analysis, we used the reconstructed single-cell phylogenies from the original study, which were inferred with the Cassiopeia package (Jones et al., 2020). In these phylogenies, each leaf corresponds to a cell, with data corresponding to the single-cell expression profile and the tissue from which it was sampled. In the original study, we described how metastatic rates of single cells could be inferred directly from these phylogenies and combined with expression profiles to identify transcriptional regulators of this process.

In the present analysis, we evaluated a clone of 1,127 cells using the PhyloVision pipeline (see Data and code availability). In this case study, we used signatures downloaded from MSigDB (Subramanian et al., 2005) and focused on the results from the Hotspot analysis. Hotspot identifies three non-overlapping modules of genes (Figure 3A). To evaluate the cumulative activity of each module at each cell in the dataset, we computed module scores for every cell using the signature-scoring procedure in VISION (DeTomaso et al., 2019; STAR Methods). Interestingly, when compared with the metastatic rate inferred from the phylogeny (which is provided as meta-data to this session), we observed that one module is negatively correlated (module 1; Pearson's $\rho = -0.30$; Figure 3B [left]), whereas one is positively correlated with this metastatic rate (module 2; Pearson's $\rho = 0.26$; Figure 3B [middle]); module 3 does not correlate with the metastatic rate in either direction (Pearson's $\rho = 0.07$; Figure 3B [right]). These results, therefore, point to candidate transcriptional programs (each represented by a module) that are heritable and are associated with different metastatic abilities.

To interpret the biological signal intrinsic to these Hotspot modules, we first projected the single-cell transcriptomic profiles onto two dimensions using Uni-form Mani-fold Approximation and Projection (UMAP; McInnes et al., 2018) and overlaid the Hotspot modules scores (Figures 3C–3E). Upon inspection, we observed that the Hotspot modules localized to distinct regions of the two-dimensional projection and therefore marked different cellular states. We observed a similar pattern, on the phylogenies themselves, with each Hotspot module marking a specific set of subclades.

Next, we examined which gene signatures had significant overlap with each module. In the module negatively correlated with metastatic rate (module 1), we found significant enrichments corresponding to the innate immune response, maintenance of the gastrointestinal epithelium, and the endosomal vacuolar pathway, among others (all FDR $< 1e-3$, hypergeometric test; Figure 3C). Together, these gene sets indicate that fewer metastatic cells in this clone are characterized by antigen presentation and maintenance of an epithelial-like state, supporting the hypothesis that tumor progression is required for metastatic competency in lung adenocarcinoma (Caswell et al., 2014).

On the other hand, the module positively correlated with metastatic rate (module 2) had significant enrichments in gene signatures associated with fibrinolysis, UV response, smooth-muscle adhesion, inflammatory response, and other metastatic signatures (e.g., liver cancer metastasis; all FDR <0.05, hypergeometric test; Figure 3D). These gene signatures therefore point to several mechanisms of enhancing metastatic rates in this clone, such as fibrinolysis and up-regulation of adhesion molecules. The diversity of these signatures underscores the importance of inflammatory signaling, ECM remodeling, and cell adhesion discussed in our previous work (Quinn et al., 2021).

Module 3 was characterized by a set of gene signatures associated with tumor progression but distinct from the metastatic rate. Intriguingly, the gene sets that significantly overlapped with module 3 included those that regulated apoptosis and

CellPress
OPEN ACCESS

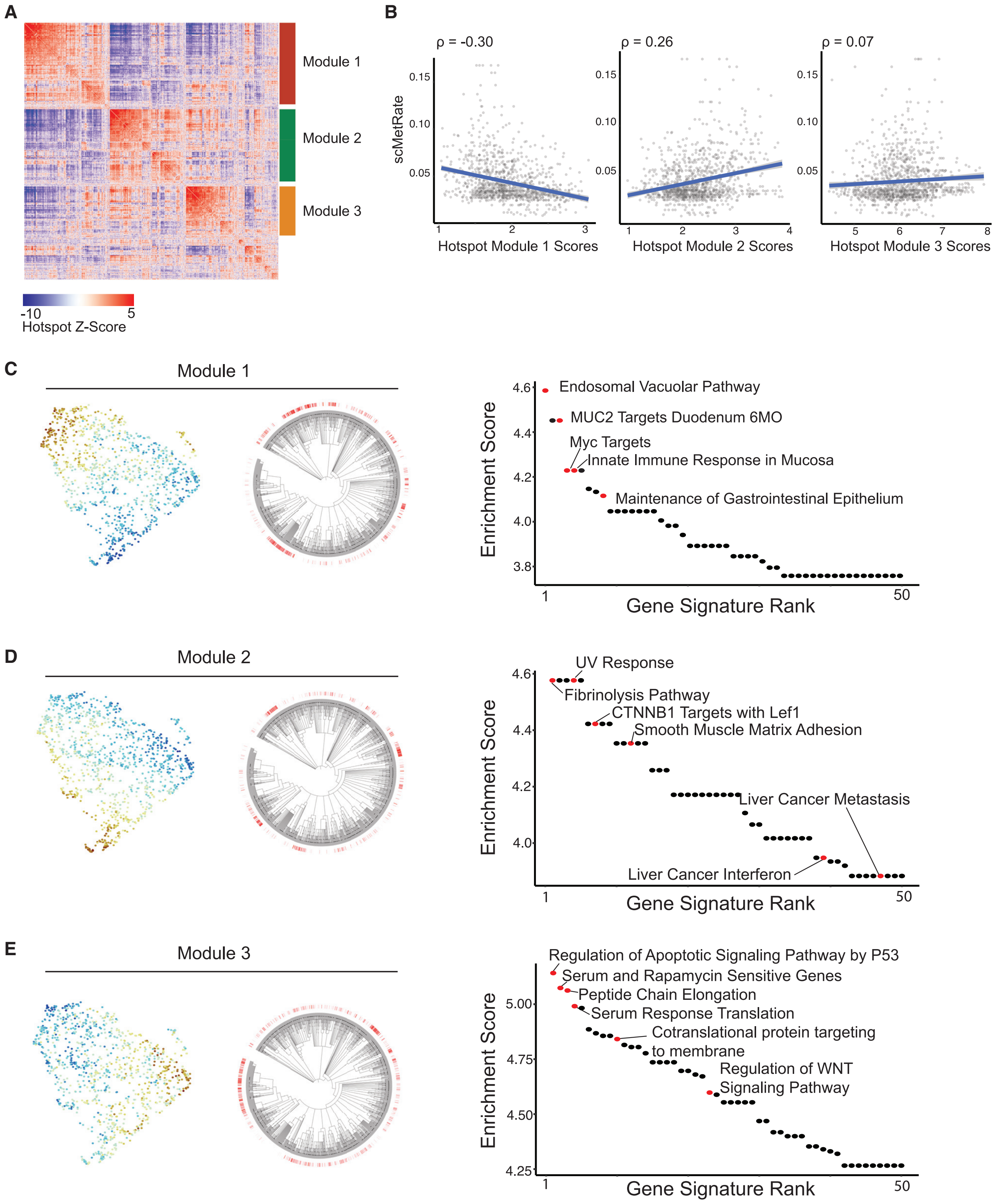

(legend on next page)

WNT-signaling, as well as those indicating sensitivity to serum and rapamycin treatment (all FDR < 1e−3, hypergeometric test; Figure 3E). These observations suggest that module 3 distinguishes cells in an altogether different metabolic state uncorrelated with metastatic ability but appearing to be associated with increased survival ability and cell proliferation, perhaps due to relatively high amounts of *KRAS* signaling. These results also support the finding that combination therapies involving inhibitors of the mammalian target of rapamycin (mTOR), which sits downstream of *RAS* signaling, is a viable therapeutic opportunity in *KRAS*-driven non-small-cell lung cancer (Vasan et al., 2014). Future work may investigate whether this therapy-sensitive population is mainly characterized by differential metabolic signaling, perhaps leveraging recent work modeling metabolic profiles from scRNA-seq data (Wagner et al., 2021).

Overall, these results indicate that the PhyloVision joint analysis of single-cell expression and lineage provides an efficient approach for dissecting phylogeny-based transcriptional heterogeneity. In the case study above, we demonstrate that the analysis pipeline and web-based user interface were able to identify gene programs associated with increased or decreased metastatic ability and altogether new programs not previously described in the original study. As a whole, this analysis provides testable hypotheses and intricate molecular characterizations for subpopulations in a single clone.

DISCUSSION

We introduced PhyloVision, a tool for the integrated analysis of scRNA-seq and single-cell lineage-tracing data. PhyloVision offers a feature-rich, user-friendly, and interactive web report for exploring the evolutionary underpinnings of scRNA-seq profiles. Moreover, PhyloVision is embedded within useful analysis pipelines, thus enabling rapid characterization of interesting structure in the high-dimensional data. In this way, to the best of our knowledge, PhyloVision is the first interactive tool of its kind to provide a bridge between single-cell analysis tools and single-cell lineage-tracing data. We show the effectiveness of this approach in a case study of a single clone from our recent work, illustrating rich heterogeneity in this dataset previously unexplored.

In addition to the Hotspot analysis presented here, PhyloVision includes other analysis features such as identification of heritable meta-data (e.g., metastatic rate) and its transcriptional correlates (using gene signatures and Hotspot modules), identification of differentially expressed genes (e.g., by manually choosing subclones to be compared), and visualization of gene expression and meta-data while stratifying the cells according to the phylogeny (e.g., with histograms corresponding to different clades).

While the case study presented in this work focused on CRISPR-Cas9-based lineage tracing, we expect that this tool will also be useful for any type of data that jointly measure cell lineage and transcriptomic state such as those from B cell phylogenies (inferred from their antigen receptor) or single-cell phylogenies built from whole-genome sequencing of tumor samples. Moreover, the shareability of generated web-based reports allows collaborators without computational experience to smoothly explore high-dimensional datasets. All of that taken together, we anticipate that PhyloVision will be useful across applications and technologies and will provide critical support for the interpretation of these multimodal datasets.

Limitations of the study

Our implementation of PhyloVision provides several opportunities for future development. First, there are many useful approaches for quantifying additional cellular characteristics from phylogenies, such as relative fitness, that are not directly supported within our software. Future efforts to incorporate these approaches into the PhyloVision pipeline would be useful. Second, our analysis of the metastasis dataset presented in this study still lacks experimental validation to support the generated hypotheses. Finally, our implementation of PhyloVision in the R programming language may provide challenges for utilizing separate software available in Python, like Cassiopeia (Jones et al., 2020), and future efforts might focus on facilitating interoperability across these ecosystems.

STAR★METHODS

Detailed methods are provided in the online version of this paper and include the following:

- KEY RESOURCES TABLE
- RESOURCE AVAILABILITY
  - Lead contact
  - Materials availability
  - Data and code availability
- METHOD DETAILS
  - The *PhyloVision* pipeline
  - Visualization of phylogenies
  - Generating stratification (clustering) of the cells according to the phylogeny
- QUANTIFICATION AND STATISTICAL ANALYSIS
  - Phylogenetic autocorrelation analysis for gene sets (signature-centric analysis)
  - Phylogenetic autocorrelation analysis for individual genes and the identification of modules (HotSpot analysis)

Figure 3. PhyloVision analysis identifies gene modules associated with metastasis and tumor progression

(A) Heatmap of Z-normalized pairwise autocorrelations between Hotspot-selected genes, clustered into three non-overlapping modules (genes not grouped into a Hotspot gene module do not have a bar annotation). Note that pairwise autocorrelations can be interpreted as a smooth and more robust estimation of a standard pairwise correlation (DeTomaso and Yosef, 2021).

(B) Hotspot module scores and the single-cell metastasis rate (scMetRate) are plotted against one another. Pearson's correlation coefficients are indicated above each scatterplot.

(C–E) Interpretation of Hotspot gene modules for module 1 (C), module 2 (D), and module 3 (E). Module scores are overlaid onto the UMAP of scRNA-seq data and phylogenies. The enrichment score between gene signature and module gene set (defined as the ratio between their observed and expected overlap; STAR Methods) is shown for the top 50 signatures. Selected gene signatures are highlighted and annotated.

- ○ Assessment of statistical significance of the overlap between Hotspot modules and user-provided gene signature
- ○ Analysis of metadata
- ○ Single-cell plasticity analysis
- ○ Differential expression analysis
- ○ Analysis of the lung cancer data

SUPPLEMENTAL INFORMATION

Supplemental information can be found online at https://doi.org/10.1016/j.crmeth.2022.100200.

ACKNOWLEDGMENTS

The authors would like to thank Alex Khodaverdian, Richard Zhang, Sebastian Prillo, Joseph Min, David DeTomaso, and the members of the Yosef lab for their helpful discussions in the development of this project. The authors would also like to thank Galen Xing, Liron Grossman, and Shirley Hui for helpful testing and feedback of the software. This work was supported by UCSF Discovery Fellowship (to M.G.J.) and NIH-NIAID U19AI090023 (to N.Y.). N.Y. is also a member of the Chan Zuckerberg Biohub investigator program.

AUTHOR CONTRIBUTIONS

All authors contributed to the design of software and analysis. N.Y. and M.G.J. conceived of this project, and N.Y. directed the work. Y.R. and M.G.J. wrote the software. M.G.J. performed the analysis on the xenograft cancer dataset. All authors wrote and approved the final manuscript.

DECLARATION OF INTERESTS

N.Y. is an adviser and/or has equity in Cellarity, Celsius Therapeutics, and Rheos Medicines.

Received: October 20, 2021
Revised: February 19, 2022
Accepted: March 28, 2022
Published: April 25, 2022

REFERENCES

Benjamini, Y., and Hochberg, Y. (1995). Controlling the false discovery rate: a practical and powerful approach to multiple testing. J. R. Stat. Soc. *57*, 289–300.

Bowling, S., Sritharan, D., Osorio, F.G., Nguyen, M., Cheung, P., Rodriguez-Fraticelli, A., Patel, S., Yuan, W.-C., Fujiwara, Y., Li, B.E., et al. (2020). An engineered CRISPR-Cas9 mouse line for simultaneous readout of lineage histories and gene expression profiles in single cells. Cell *181*, 1410–1422.e27.

Caswell, D.R., Chuang, C.-H., Yang, D., Chiou, S.-H., Cheemalavagu, S., Kim-Kiselak, C., Connolly, A., and Winslow, M.M. (2014). Obligate progression precedes lung adenocarcinoma dissemination. Cancer Discov. *4*, 781–789.

Chan, M.M., Smith, Z.D., Grosswendt, S., Kretzmer, H., Norman, T.M., Adamson, B., Jost, M., Quinn, J.J., Dian, Y., Jones, M.G., et al. (2019). Molecular recording of mammalian embryogenesis. Nature *570*, 77–82.

DeTomaso, D., Jones, M.G., Subramaniam, M., Ashuach, T., Ye, C.J., and Yosef, N. (2019). Functional interpretation of single-cell similarity maps. Nat. Commun. *10*, 4376. https://doi.org/10.1101/403055.

DeTomaso, D., and Yosef, N. (2021). Hotspot identifies informative gene modules across modalities of single-cell genomics. Cell Syst. *12*, 446–456.e9.

Fitch, W.M. (1971). Toward defining the course of evolution: minimum change for a specific tree topology. Systematic Biology *20*, 406–416.

Frieda, K.L., Linton, J.M., Hormoz, S., Choi, J., Chow, K.H.K., Singer, Z.S., Budde, M.W., Elowitz, M.B., and Cai, L. (2017). Synthetic recording and in situ readout of lineage information in single cells. Nature *541*, 107–111.

Gayoso, A., Lopez, R., Xing, G., Boyeau, P., Valiollah Pour Amiri, V., Hong, J., Wu, K., Jayasuriya, M., Mehlman, E., Langevin, M., and Liu, Y. (2022). A Python library for probabilistic analysis of single-cell omics data. Nature Biotechnology *40*, 163–166.

Geary, R.C. (1954). The Contiguity ratio and statistical mapping. The Incorporated Statistician *5*, 115–146.

Hartigan, J.A. (1973). Minimum mutation fits to a given tree. Biometrics *29*, 53–65.

Jones, M.G., Khodaverdian, A., Quinn, J.J., Chan, M.M., Hussmann, J.A., Wang, R., Xu, C., Weissman, J.S., and Yosef, N. (2020). Inference of single-cell phylogenies from lineage tracing data using Cassiopeia. Genome Biol. *21*, 92.

Kalhor, R., Mali, P., and Church, G.M. (2017). Rapidly evolving homing CRISPR barcodes. Nat. Methods *14*, 195–200.

Kenyon, C.J. (2010). The genetics of ageing. Nature *464*, 504–512.

Kester, L., and van Oudenaarden, A. (2018). Single-cell transcriptomics meets lineage tracing. Cell Stem Cell *23*, 166–179.

Letunic, I., and Bork, P. (2021). Interactive Tree of Life (iTOL) v5: an online tool for phylogenetic tree display and annotation. Nucleic Acids Res. *49*, W293–W296. https://doi.org/10.1093/nar/gkab301.

Lopez, R., Regier, J., Cole, M.B., Jorden, M.I., and Yosef, N. (2018). Deep generative modeling for single-cell transcriptomics. Nat. Methods *15*, 1053–1058.

Lu, T., Aron, L., Zullo, J., Pan, Y., Kim, H., Chen, Y., Yang, T.-H., Kim, H.-M., Drake, D., Liu, X.S., et al. (2014). REST and stress resistance in ageing and Alzheimer's disease. Nature *507*, 448–454.

van der Maaten, L. (2008). Visualizing Data using t-SNE. J. Machine Learn. Research *9*, 2579–2605.

McInnes, L., Healy, J., and Melville, J. (2018). UMAP: Uniform Manifold approximation and projection for dimension reduction. Preprint at arXiv, 1802.03426.

McKenna, A., Findlay, G.M., Gagnon, J.A., Horwitz, M.S., Schier, A.F., and Shendure, J. (2016). Whole organism lineage tracing by combinatorial and cumulative genome editing. Science *353*, aaf7907.

McKenna, A., and Gagnon, J.A. (2019). Recording development with single cell dynamic lineage tracing. Development *146*, dev169730. https://doi.org/10.1242/dev.169730.

Neher, R.A., Russell, C.A., and Shraiman, B.I. (2014). Predicting evolution from the shape of genealogical trees. Elife *3*, e03568.

Packer, J.S. (2019). A lineage resolved molecular atlas of *C. elegans* embryogenesis at single-cell resolution. Science *365*, eaax1971.

Paradis, E., and Schliep, K. (2019). Ape 5.0: an environment for modern phylogenetics and evolutionary analyses in R. Bioinformatics *35*, 526–528.

Quinn, J.J., Jones, M.G., Okimoto, R.A., Nanjo, S., Chan, M.M., Yosef, N., Bivona, T.G., and Weissman, J.S. (2021). Single-cell lineages reveal the rates, routes, and drivers of metastasis in cancer xenografts. Science *371*, eabc1944.

Raj, B., Wagner, D.E., McKenna, A., Pandey, S., Klein, A.M., Shendure, J., Gagnon, J.A., and Schier, A.F. (2018). Simultaneous single-cell profiling of lineages and cell types in the vertebrate brain. Nat. Biotechnol. *36*, 442–450.

Salvador-Martínez, I., Grillo, M., Averof, M., and Telford, M.J. (2021). CeLaVi: an interactive cell lineage visualization tool. Nucleic Acids Res. *49*, W80–W85.

Satija, R., Farrell, J.A., Gennert, D., Schier, A.F., and Regev, A. (2015). Spatial reconstruction of single-cell gene expression data. Nature biotechnology *33*, 495–502.

Subramanian, A., Tamayo, P., Mootha, V.K., Mukherjee, S., Ebert, B.L., Gillette, M.A., Paulovich, A., Pomeroy, S.L., Golub, T.R., Lander, E.S., and Mesirov, J.P. (2005). Gene set enrichment analysis: a knowledge-based

approach for interpreting genome-wide expression profiles. Proc. Natl. Acad. Sci. U S A *102*, 15545–15550.

Sulston, J.E., Schierenberg, E., White, J.G., and Thomson, J.N. (1983). The embryonic cell lineage of the nematode Caenorhabditis elegans. Dev. Biol. *100*, 64–119.

Vasan, N., Boyer, J.L., and Herbst, R.S. (2014). A RAS renaissance: emerging targeted therapies for KRAS-mutated non-small cell lung cancer. Clin. Cancer Res. *20*, 3921–3930.

Wagner, A., Wang, C., Fessler, J., DeTomaso, D., Avilo-Pacheco, J., Kaminski, J., Zaghouani, S., Christian, E., Thakore, P., Schellhaass, B., et al. (2021). Metabolic modeling of single Th17 cells reveals regulators of autoimmunity. Cell *184*, 4168–4185.

Wagner, D.E., and Klein, A.M. (2020). Lineage tracing meets single-cell omics: opportunities and challenges. Nat. Rev. Genet. *21*, 410–427.

Yang, D., Jones, M.G., Naranjo, S., Rideout, W.M., Min, K.H.J., Ho, R., Wu, W., Replogle, J.M., Page, J.L., Quinn, J.J., and Horns, F. (2021). Lineage Recording Reveals the Phylodynamics, Plasticity and Paths of Tumor Evolution. Preprint at bioRxiv. https://doi.org/10.1101/2021.10.12.464111.

CellPress
OPEN ACCESS

STAR★METHODS

KEY RESOURCES TABLE

REAGENT or RESOURCE	SOURCE	IDENTIFIER
Deposited data		
Embyrogenesis PhyloVision report	Chan et al. 2019 (GEO Accession GSE117542)	Zenodo: https://doi.org/10.5281/zenodo.6354746
Metastasis PhyloVision report	Quinn et al. 2021 (GEO Accession GSE161363)	Zenodo: https://doi.org/10.5281/zenodo.6354746
Software and algorithms		
PhyloVision	This study	Zenodo: https://doi.org/10.5281/zenodo.3345984

RESOURCE AVAILABILITY

Lead contact

Further information and requests for resources should be directed to and will be fulfilled by the lead contact Nir Yosef (niryosef@berkeley.edu).

Materials availability

This study did not generate new unique reagents.

Data and code availability

- This paper analyzes existing, publicly available data. These accession numbers for the datasets are listed in the key resources table. Interactive *PhyloVision* reports are deposited on Zenodo and are publicly available as the date of publication. DOIs are listed in the key resources table.
- All original code is available on Github (https://github.com/Yoseflab/VISION) and on Zenodo and is publicly available as of date of the publication. DOIs are listed in the key resources table.
- Any additional information required to reanalyze the data reported in this paper is available from the lead contact upon request.

METHOD DETAILS

The *PhyloVision* pipeline

PhyloVision builds on the *VISION* analysis toolkit for signature autocorrelation analysis (DeTomaso et al., 2019). As input, *PhyloVision* requires a gene expression matrix (typically count-normalized, but not log-normalized), a set of signature gene sets (e.g., publicly available from sources like MSigDB), and a phylogeny (stored as a tree structure in the *ape* R package (Paradis and Schliep, 2019)). Amongst other data that can be optionally passed into *PhyloVision* are numerical and categorical metadata, as well as a two-dimensional projections of the cells for visualization purposes (e.g. from t-distributed stochastic neighbor embedding [tSNE] (van der Maaten, 2008) of the main principal components or of an embedding learned by methods such as scVI (Lopez et al., 2018; Gayoso et al., 2022). In the original VISION pipeline, cell-level clustering and consistency evaluation was performed on a user-specified "latent space" (a low-dimensional embedding such as the top principal components or an embedding inferred with tools like scVI). In the PhyloVision pipeline the phylogeny over all cells is treated as this latent space, and all clustering and consistency analysis is performed using the cell-cell similarities encoded by the phylogeny (to see how cell clusters are utilized, see section "Analysis of metadata"). Optionally, a user can still specify an additional latent space that will be used for inferring additional single-cell visualizations using algorithms like tSNE and UMAP. Finally, users can also specify additional pre-computed Hotspot objects that can be used for the Hotspot analysis (as described below).

The signature-centric (VISION) part of the analysis pipeline in *PhyloVision* (invoked by default when running *PhyloVision*) begins by clustering cells according to substructure on the tree (see below, section "Generating stratification (clustering) of the cells according to the phylogeny") and computing scores for each user-provided signature and for every cell (computed using cell-level Z-scores as in (DeTomaso et al., 2019)). It then assesses the agreement between the signature scores and the phylogeny with a phylogenetic autocorrelation statistic (see section entitled "Phylogenetic autocorrelation analysis"). Signatures that are significantly auto-correlated (i.e., cells that are nearby in the phylogeny have more similar scores than expected by chance) are clustered using the previously described *VISION* pipeline (DeTomaso et al., 2019) and displayed in the web-based report in a collapsable table for each cluster

(Figure 1, inset 3). The user can explore the scores of these gene signatures by overlaying their scores on the two-dimensional representation of the gene expression data (Figure 1, inset 1) and the phylogeny (Figure 1 inset 2), viewing histograms of these scores possibly subsetted according to the phylogeny (Figure 1, inset 4), and browsing through the respective genes (Figure 1 panel 4; where genes are ranked their covariance with the signature score).

As with VISION, cell-level metadata is handled similarly to signature scores computed within PhyloVision. Specifically, separate methodology is used to infer autocorrelation of numerical and categorical metadata. However, PhyloVision builds on this autocorrelation analysis by providing utilities for quantifying cell-level plasticity scores for each categorical metadata item. Intuitively, these scores represent how often a categorical variable transitioned between states along the phylogeny (for example, the scMetRate from Quinn et al., 2021). For more details, refer to the sections below "Analysis of metadata" and "Single-cell plasticity analysis".

PhyloVision additionally supports a phylogenetic analysis with *Hotspot*, which can be invoked with the *runHotspot* function. This function interfaces with the *Hotspot* tool, implemented in Python, using the *reticulate* R package. Using the previously described -*Hotspot* pipeline (DeTomaso and Yosef, 2021)*, PhyloVision* first identifies individual genes whose expression is coherent with the tree structure (i.e., nearby cells in the tree express the gene at a similar level, as compared to random chance). It then performs a two-dimensional autocorrelation analysis to group the identified genes into modules. Finally, *PhyloVision* computes an enrichment statistic between user-defined gene signatures and the identified *Hotspot* modules (see section entitled "Assessment of statistical significance of the overlap between Hotspot modules and user-provided gene signature"). Gene signatures with significant overlap are included in the web-based report in a collapsable table for each *Hotspot* module (Figure 1, inset 3). The user can explore the scores of these gene signatures or the score of entire hotspot modules by overlaying their cumulative expression (computed identically to user-specified signatures, as in (DeTomaso et al., 2019)) on the 2-dimensional representation of the gene expression data (Figure 1, inset 1) and the phylogeny (Figure 1, inset 2), viewing histograms of these scores possibly subsetted according to the phylogeny (Figure 1, inset 4), and browsing through the respective genes (Figure 1, panel 4; where genes are ranked by covariance as with user-specified signatures).

Visualization of phylogenies

PhyloVision's web-based user interface displays the provided phylogeny using a custom Plotly Javascript package (PhyloPlot.js) in either a radial or linear layout. Users are able to select individual cells and clades on the phylogeny for use in Differential Expression and to view on the UMAP. Leaves reflect the same cell-coloring as the UMAP. Users are able to collapse clades to summary nodes, using the mode, arithmetic mean, geometric mean or median of the numerical values selected for the node's leaves. Users can also collapse nodes by depth from the root of the tree. The phylogeny in both radial and linear layouts is converted to ultrametric edge lengths using the following formula:

$$depth(node) = \{1 \; if \; node \; is \; leaf; else \; \max(depth(child)) = 1 \; \forall children(node)\}$$

Generating stratification (clustering) of the cells according to the phylogeny

In many cases, a phylogeny can be used to stratify groups of cells into specific groups by "cutting" the tree at a specific depth and assigning cells to subclonal lineages. For example, if a phylogeny describes a differentiation process between several cell types, a clustering of cells into subclades might yield cell-type specific clusters. PhyloVision performs not only a clustering of the phylogeny but also an assessment of how meaningful the clustering is on the tree via an autocorrelation statistic (representing how similar the cluster assignment is of neighboring cells).

PhyloVision clusters cells on a phylogeny by performing a breadth-first search over internal nodes. Specifically, the algorithm maintains a queue of internal nodes and updates the queue by popping off the internal node with the largest child clade size, and adding it's immediate children to the queue. This algorithm begins with the root node, and terminates once the queue has reached a target length (defaulted to 10) at which point *PhyloVision* merges the smallest clade with its neighbor until the exact target number is reached. The algorithmic pseudocode is detailed as follows:

```
Cluster-Phylogeny (target := 10)
    Queue := Priority Queue {Node : Maximum size of Node's child clades}
    Insert the root into the queue
    While length(queue) <= target do:
        Node := queue.pop(1)
        Insert Node's children to Queue
    Clusters := {[Children]} Nodes Queue
    While length(Clusters) > target do:
        Select smallest cluster from Clusters
        Merge that cluster with its phylogenetically nearest cluster
    Return Clusters
```

While these clusters can be accessed via the metadata variable "VISION_Clusters_Tree", additional clusterings derived from the phylogeny may be computed and stored as metadata (see *PhyloVision* vignette available with the package (https://yoseflab.github.io/VISION/articles/phyloVision.html)).

QUANTIFICATION AND STATISTICAL ANALYSIS

Phylogenetic autocorrelation analysis for gene sets (signature-centric analysis)

To compute the extent to which a value (e.g., signature score, module score, or continuous covariate) can explain the cellular relationships on the phylogeny, we make use of the Geary's *C* statistic for local autocorrelation. This statistic is defined as

$$C = \frac{(N-1)\sum_i \sum_j w_{ij}(x_i - x_j)^2}{2W\sum_i (x_i - \bar{x})^2}$$

where w_{ij} represents the cophenetic distance (i.e., distance between cells using the branch lengths of the user-specified phylogeny) between cells *i* and *j*, x_i is a value of interest, *N* is the total number of cells, and *W* is the sum of all weights. (A small amount of random noise is introduced to the cophenetic distances to break ties.) In our case, the value of interest (i.e. *x*) are the ranks of the normalized signature score in each cell, as defined previously (DeTomaso et al., 2019). We report $C' = 1 - C$ such that a score of 1 indicates perfect autocorrelation and 0 means no autocorrelation. While the C' statistic provides an effect size, we evaluate the significance of gene signature scores with an empirical p-value (FDR corrected with the Benjamini-Hochberg procedure (Benjamini and Hochberg, 1995)), comparing the signature score to a background of randomly generated signatures as described in our previous work (DeTomaso et al., 2019). In the collapsable tables displayed on the UI (Figure 1, inset 3), we retain only gene signatures with an FDR <0.05 and report both C' statistics and the FDR.

In the phylogenetic autocorrelation analysis, we utilize a K-nearest neighbor (KNN) graph where weights are only non-zero between a cell *i* and it's closest *k* neighbors. Specifically, if cell *j* is not a k-nearest neighbor of cell *i*, then w_{ij} is taken to be 0. K-nearest neighbors of cell *i* are found using the distances on the phylogeny, where the distance between cells *i* and *j* is defined as the sum of the edge lengths on the path between the two cells. If edge lengths for the dendrogram are not provided, every edge is defaulted to length 1. Ties are randomly broken.

Phylogenetic autocorrelation analysis for individual genes and the identification of modules (HotSpot analysis)

PhyloVision employs a gene-level clustering into modules using the *Hotspot* autocorrelation analysis (DeTomaso and Yosef, 2021) on the user-defined phylogeny. (Here we describe how the algorithm is applied in the *PhyloVision* pipeline, for mathematical details please refer to our previous work (DeTomaso and Yosef, 2021).) First, using the cophenetic distances on the tree (defined as the phylogenetic distance separating cells), a *K*-nearest neighbor (KNN) graph is constructed (using a default $K = sqrt(N)$, where *N* is the number of cells). Then, genes are selected that are significantly autocorrelated with the phylogenetic KNN graph using the "*compute_autocorrelations*" function in *Hotspot*. By default, the depth-adjusted negative binomial ("danb") model is used and the top 1000 genes are selected (as measured by *Hotspot's* Z-transformed Geary's C) that pass an 0.05 FDR threshold, though both these parameters can be controlled by the user. Then, genes are grouped into modules by clustering the pairwise autocorrelation matrix computed with *Hotspot's "compute_local_correlations"* function. By default, we use a minimum gene threshold of 20 and a clustering FDR of 0.5, though both parameters are controllable by the user. Gene signatures corresponding to the genes in a module are added to the *PhyloVision* object. Additionally, for each module and user-specified gene-signature pair, a new signature is created by computing the gene overlap and added to the *PhyloVision* object.

Assessment of statistical significance of the overlap between Hotspot modules and user-provided gene signature

Given a full set of *N* genes, *PhyloVision*'s compares the genes in module set *M* identified by *Hotspot* and the genes in an existing signature set *S* by first computing an enrichment statistic:

$$Overlap(M, S) = |M \cap S|$$

$$E[Overlap(M, S)] = \frac{|M| * |S|}{N}$$

$$Enrichment(M, S) = \log\left(\frac{Overlap(M,S)}{E[Overlap(M,S)]}\right)$$

Then, we assess significance using a hypergeometric test with *R*:

$$m = Max(|M|, |S|)$$

$$k = Min(|M|, |S|)$$

$$n = N - m$$

$$P(M,S) = 1 - phyper(Overlap(M,S) - 1,\ m,\ n, k)$$

where $P(M,S)$ indicates the p-value of the overlap. P-values are then FDR-corrected using the Benjamini-Hochberg procedure (Benjamini and Hochberg, 1995).

Analysis of metadata

PhyloVision utilizes the tools in *VISION* to conduct autocorrelation analysis on metadata. In this, *PhyloVision* analyzes metadata differently depending on if is numerical (i.e., continuous covariates like the number of genes detected in a cell) or categorical (i.e., discrete covariates like the batch in which a cell profile was sampled). While most metadata is specified by the user, *PhyloVision* additionally clusters the cells into subclades using a tree-based clustering method (see section below entitled "Generating stratification (clustering) of the cells according to the phylogeny").

In the context of numerical metadata, *PhyloVision* utilizes an approach to assess autocorrelation and significance identical to that of gene-signatures (see above, "Phylogenetic autocorrelation analysis for gene sets (signature-centric analysis), with the exception that scores need to be computed as they are provided as precomputed scores by the user.

In the context of categorical data, *PhyloVision* uses the Cramer's V statistic as an autocorrelation statistic. As described in our previous work (DeTomaso et al., 2019), the Cramer's V statistic is a transformation of a chi-squared test statistic, computed on the local neighborhood of each cell. Specifically, for each cell i, we compute a local proportion of each variable value m across its K phylogenetic neighbors (indexed by j):

$$\widehat{c_{im}} = \sum_j w_{i,j} I_m(c_j)$$

where w_{ij} are computed as above in the gene-centric analysis (see section "Phylogenetic autocorrelation analysis for gene sets (signature-centric analysis)"), c_j represents the value of the discrete variable of interest in cell j and $I_m(x)$ is an indicator function that takes on the value of 1 if $x == m$ and 0 otherwise. From these values, a contingency table X is computed as

$$X_{lm} = \sum_i \widehat{c_{im}} I_l(c_i)$$

The chi-squared test is then performed on this contingency table X to estimate a p-value. From the chi-squared test statistic, t, the Cramer's V statistic is computed as

$$V = sqrt\left(\frac{t}{n\ \times \min(N-1,\ M-1)}\right)$$

where n is the sum of all the values in the contingency table X, N is the number of rows in the contingency table and M is the number of columns.

Single-cell plasticity analysis

PhyloVision enables users to quantify the plasticity of a particular categorical metadata for each leaf on the tree using previously described methodology (Quinn et al., 2021; Yang et al., 2021). In this, plasticity indicates how often a given cell's ancestors changed between categories (e.g., cellular states). Specifically, this plasticity analysis begins with computing the small parsimony of a particular categorical metadata using the Fitch-Hartigan algorithm (Fitch, 1971; Hartigan, 1973) for each subtree contained within a tree (where a subtree is defined as the set of nodes downstream from a particular internal node). Each of these parsimony scores is normalized to the number of edges in the subtree. Then, the single-cell plasticity score for each cell is defined as the average of these normalized parsimony scores for all the subtrees that contain this particular leaf. This is accomplished by performing a depth-first-traversal from the root to a leaf, averaging the normalized parsimony scores of each internal node along the way.

Differential expression analysis

Differential Expression is performed using one of several tests on the gene-level expression between two groups of cells chosen by the user. By default, a Wilcoxon Rank Sums test is used, though PhyloVision also supports other tests implemented in the Seurat package such t-test and logistic regression (Satija et al., 2015). The cell groups can be chosen from user-provided metadata factors such as sample, tissue or cluster, or UI selections on the UMAP or phylogeny. Users can select cells on the UMAP using a box select or lasso select. Users can select cells on the phylogeny using a lasso select, or by choosing the parent internal node of the cells they wish to include. Users can compare metadata groups or selections to the remaining unselected cells, or to other groups or selections. The log fold change, AUC and FDR-adjusted p value are reported for each gene in the dataset.

Analysis of the lung cancer data

The tumor phylogeny for CP004 was reconstructed using the Cassiopeia-Hybrid (Jones et al., 2020) algorithm from processed single-cell lineage tracing data, as described in the original study (Quinn et al., 2021). Cells present in both the expression matrix and the lineage for CP004 were used; otherwise, cells were pruned from the lineage using the *ape* R package (Paradis and Schliep, 2019) or removed from the expression matrix. All unifurcations (i.e., nodes containing exactly one child) were collapsed using the *collapse.singles* function in the R package *ape.* Before analysis with *PhyloVision*, the cells of expression matrix of raw UMI counts were library-size-normalized to the median number of UMIs in CP004. Informative genes were found using the *filterGenesFano* function in VISION with default parameters and passed to *PhyloVision* via the *projection_genes* parameter. Signatures were downloaded from the MSigDB database (https://www.gsea-msigdb.org/gsea/msigdb/) and the Hallmark, C2, and C5 (BP) collections were used for analysis. Meta data corresponding to each cell was downloaded from NCBI GEO, series GSE161363 and dataset GSM4905334. *PhyloVision* was run with default parameters, except for setting *num_neighbors = 30* and *projection_methods = c(“UMAP”, “tSNE30”)*. After *PhyloVision* analysis, *Hotspot* was invoked using the *runHotspot* command with the following parameters: *model = “normal”, tree=TRUE, min_gene_threshold=70, n_neighbors = 30, number_top_genes = 1000*. Reported pairwise gene autocorrelations, gene modules, and enrichment results were generated from the *PhyloVision* pipeline as described above.

Cell Reports Methods, Volume *2*

Supplemental information

Interactive, integrated analysis of single-cell transcriptomic and phylogenetic data with PhyloVision

Matthew G. Jones, Yanay Rosen, and Nir Yosef

SUPPLEMENTARY FIGURES AND LEGENDS

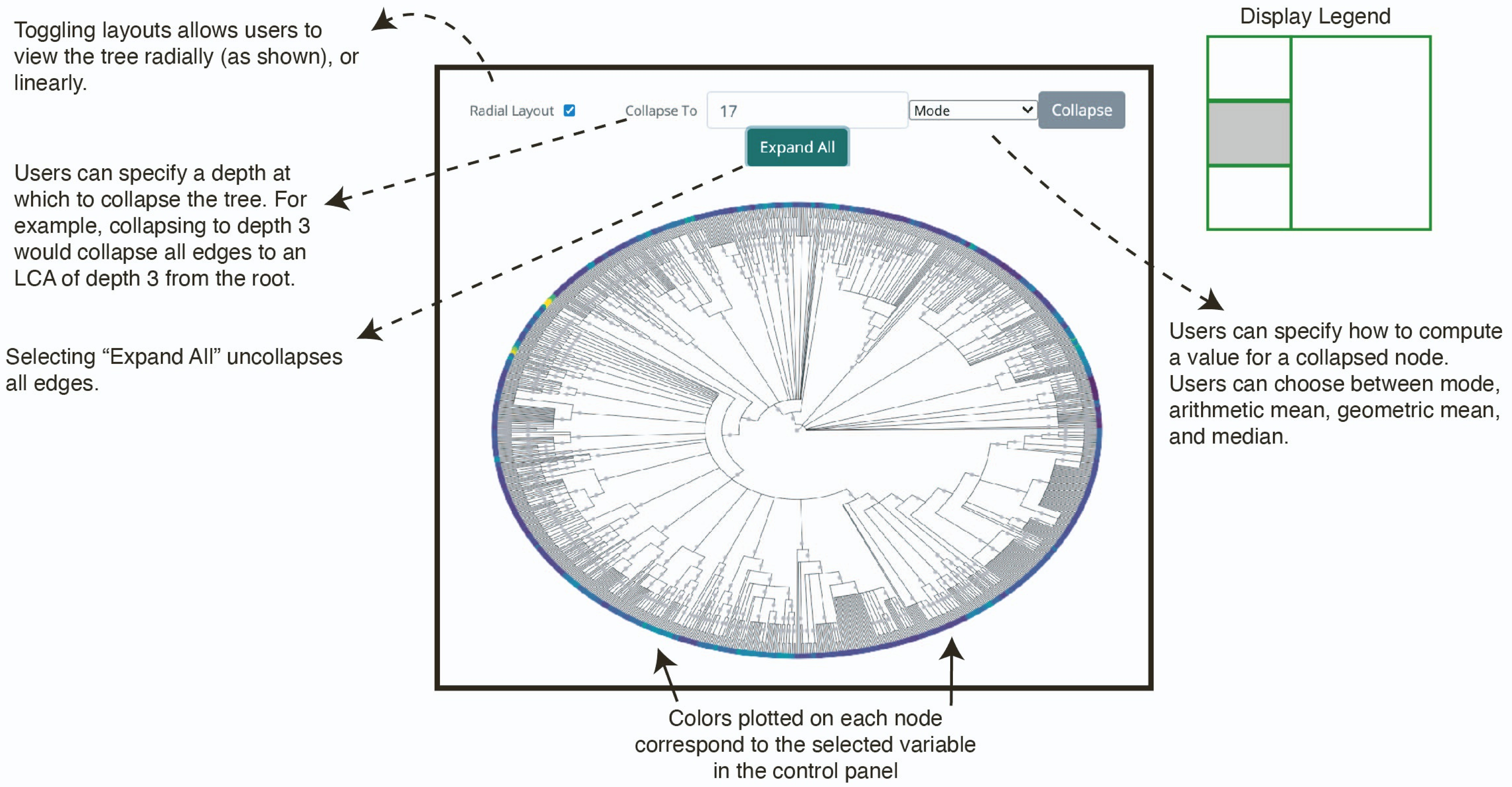

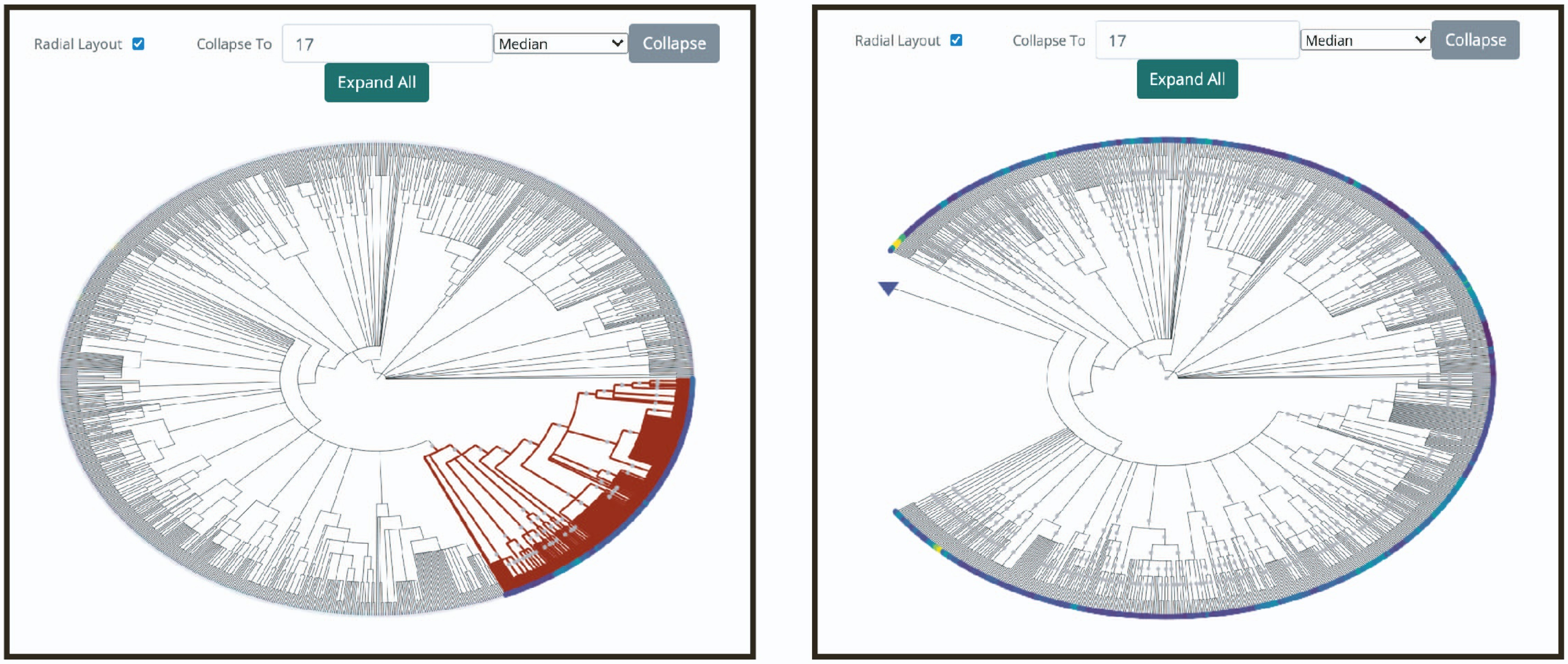

Clades can be selected by clicking with the left-mouse button on an internal node. These selections can be propagated to the cell projection viewer or the differential expression analysis tool.

Clades can be collapsed individually by clicking with the right-mouse button on an internal node. Values displayed will be computed using the specified function (e.g., median)

Figure S1: Elements of the PhyloPlot interface, related to Figure 1. A tutorial over the features of the PhyloPlot.js interface used in the *PhyloVision* web-based report. Screenshots indicate how to interact with the object, including how to select, collapse, and expand clades.

“Hotspot” mode can be selected in the top right corner of the web-report

Display Legend

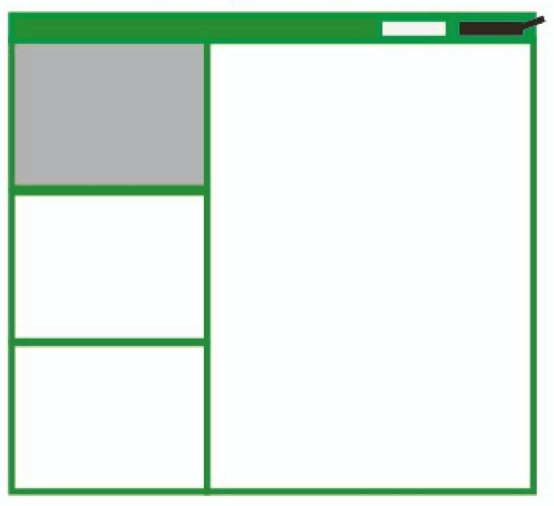

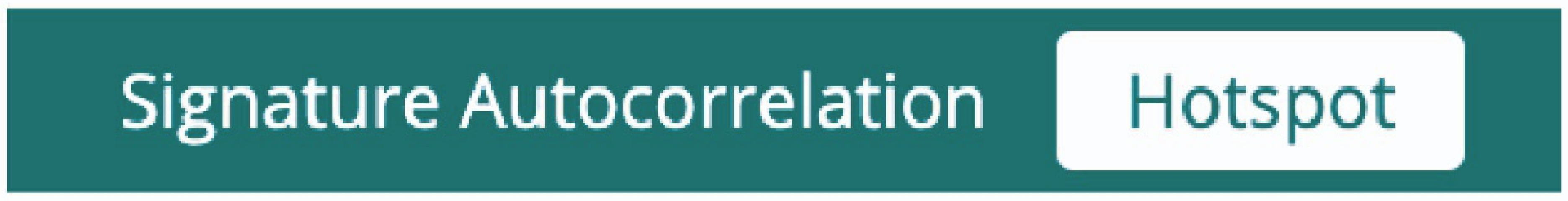

The values in the first column, labeled “Overlap” display the enrichment statistic between the genes in the Hotspot module and signature

The control panel in “Hotspot” mode contains drop down panels for each Hotspot gene module identified.

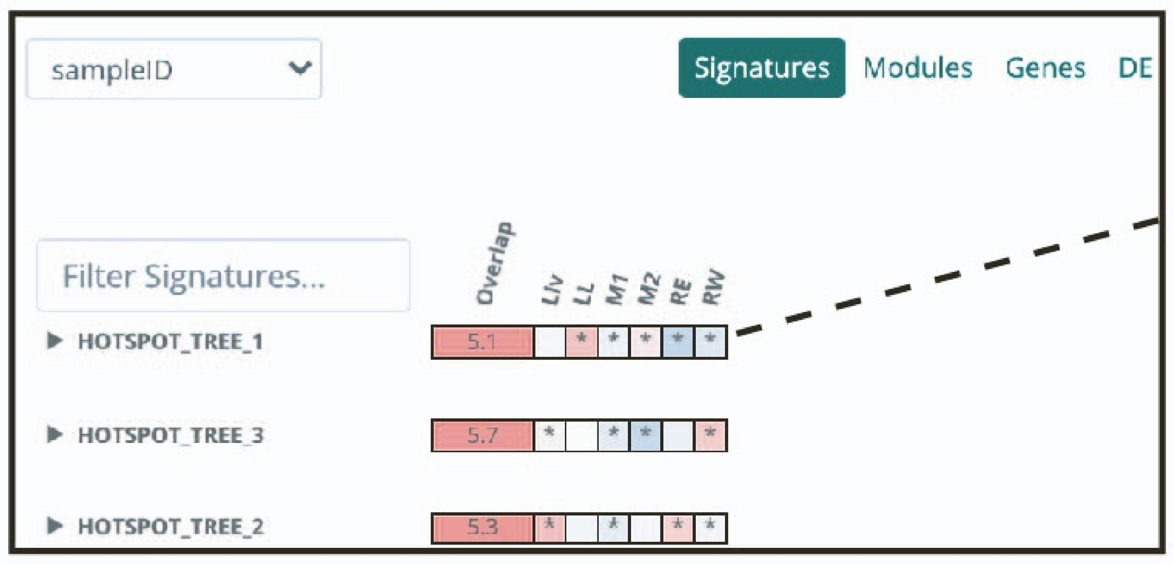

Clicking on the arrow next to a module reveals a table with entries for each signature that significantly overlaps with the module

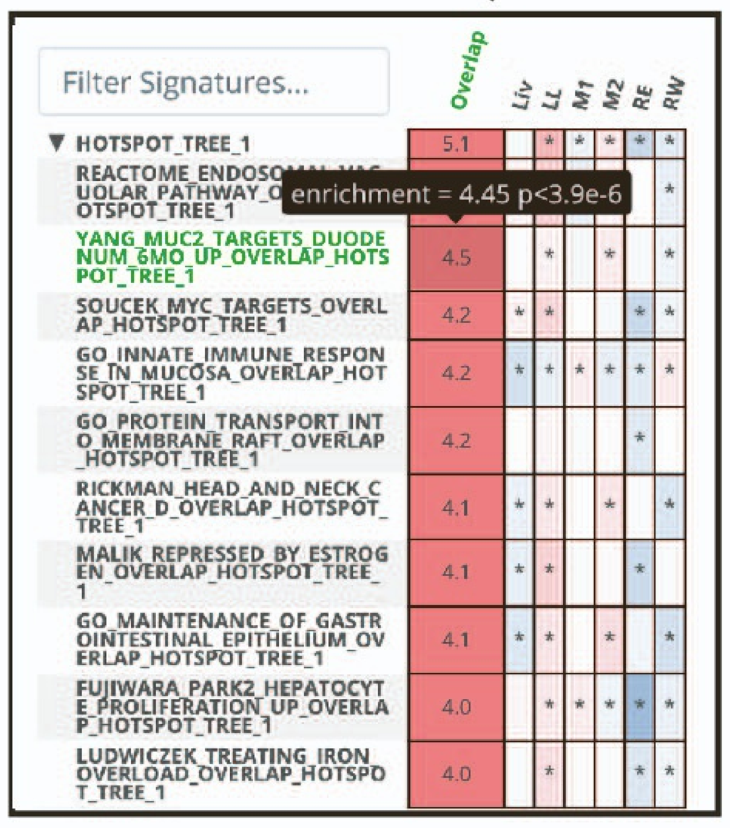

Clicking on a row displays the signature score on the cell-projection and tree. In Hotspot mode, signature scores are computed on the overlapping genes.

Hovering over a cell dispalys its enrichment statistic and FDR-corrected p-value.

Additional columns show the result of 1 vs. All differential signature tests for the selected grouping variable.

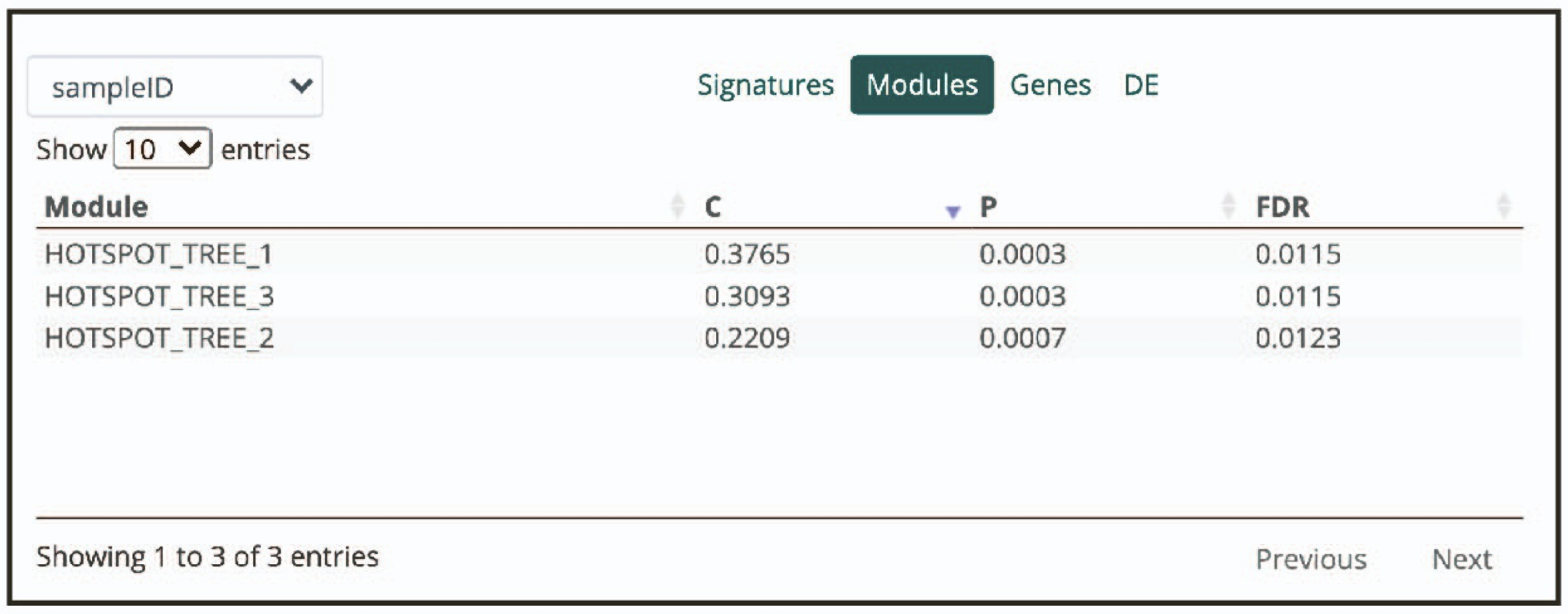

Hotspot module statistics can be viewed under the “Modules” tab.

The Geary’s C, p-value, and FDR value are displayed for each module.

The number of modules per page can be selected.

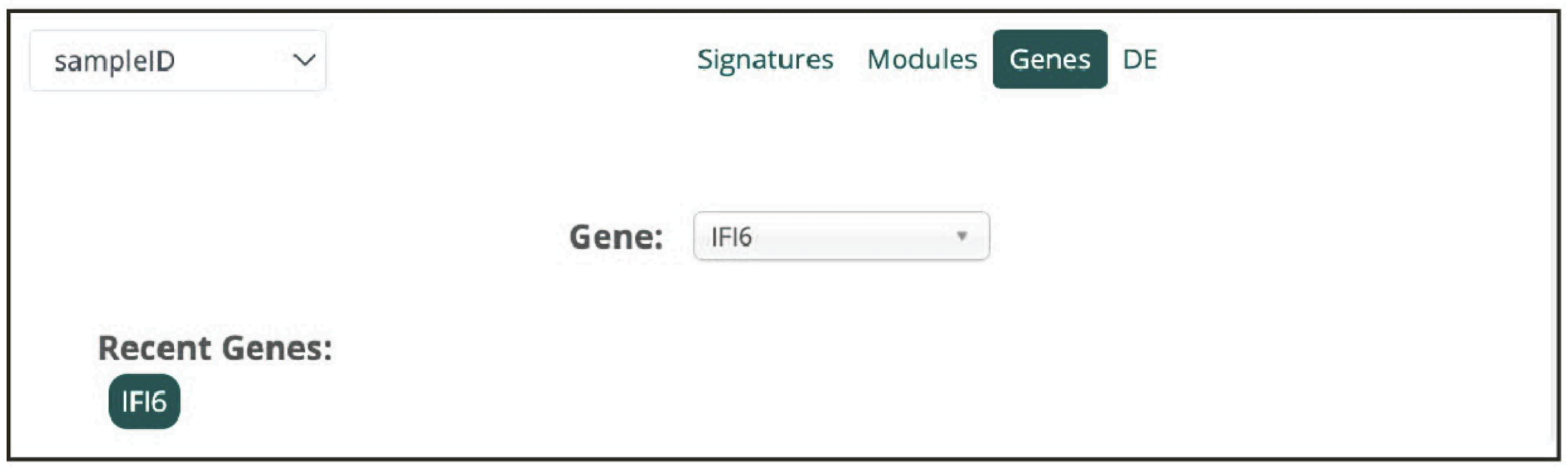

Gene expression can be overlaid onto the tree and two-dimensional embedding.

Genes can be selected via the dropdown menu.

Recently used genes can be selected under the “Recent Genes” list.

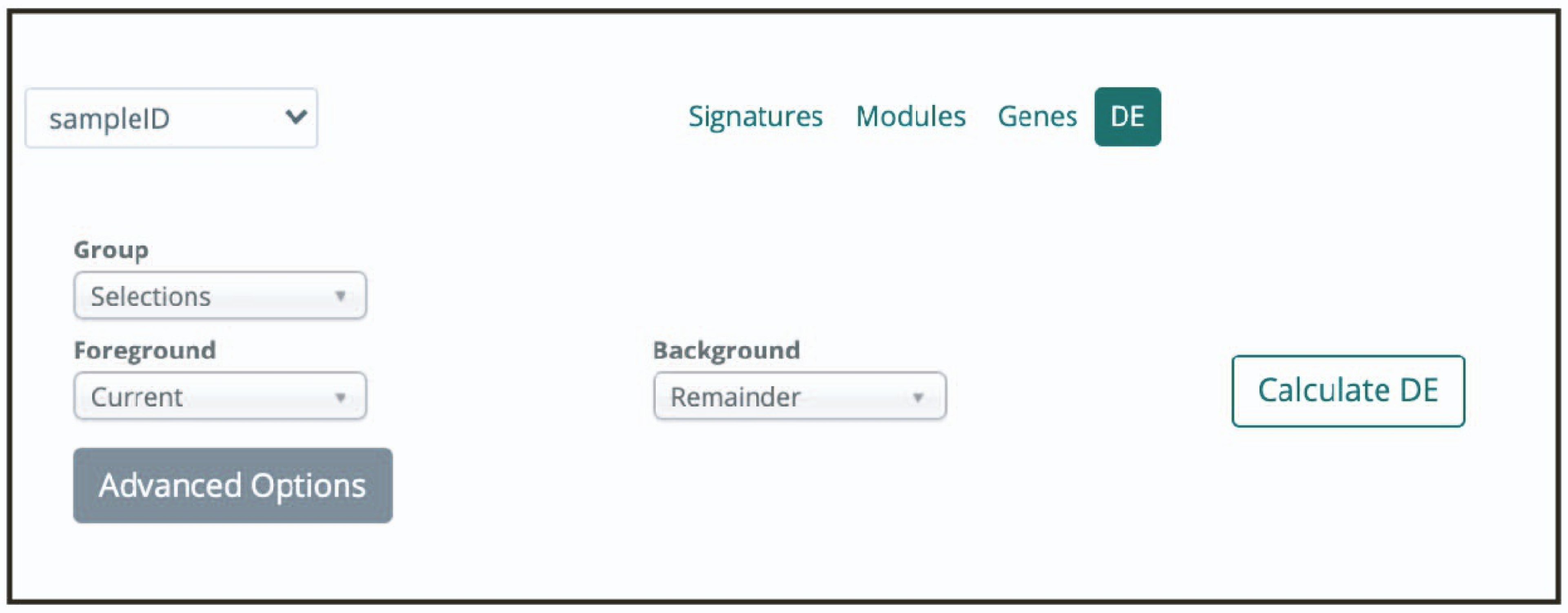

A differential expression anlaysis can be conducted under the “DE” tab. Various tests can be selected under “Advanced Options.”

A selected group can be compared against another group, or the remainder of cells if no group is specified.

Figure S2: Elements of the *Hotspot* mode control panel, related to Figure 1. Users can toggle between signature autocorrelation and *Hotspot* modes in the top-right corner of the web-based report. In *Hotspot* mode, each module is presented in a collapsable table. Elements in the table are gene signatures that significantly overlap with the *Hotspot* module (see Methods). Users can also view the *Hotspot* summary statistics with the "Modules" tab, where the Geary's C, p-values, and FDR of each module are displayed. Users can also select specific genes for displaying on the phylogeny and scatter plot, as well as perform differential expression tests. Multiple differential expression tests are supported under "Advanced Options".

Display Legend

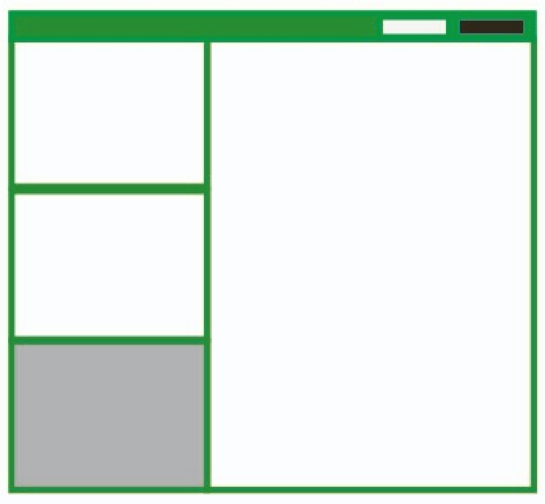

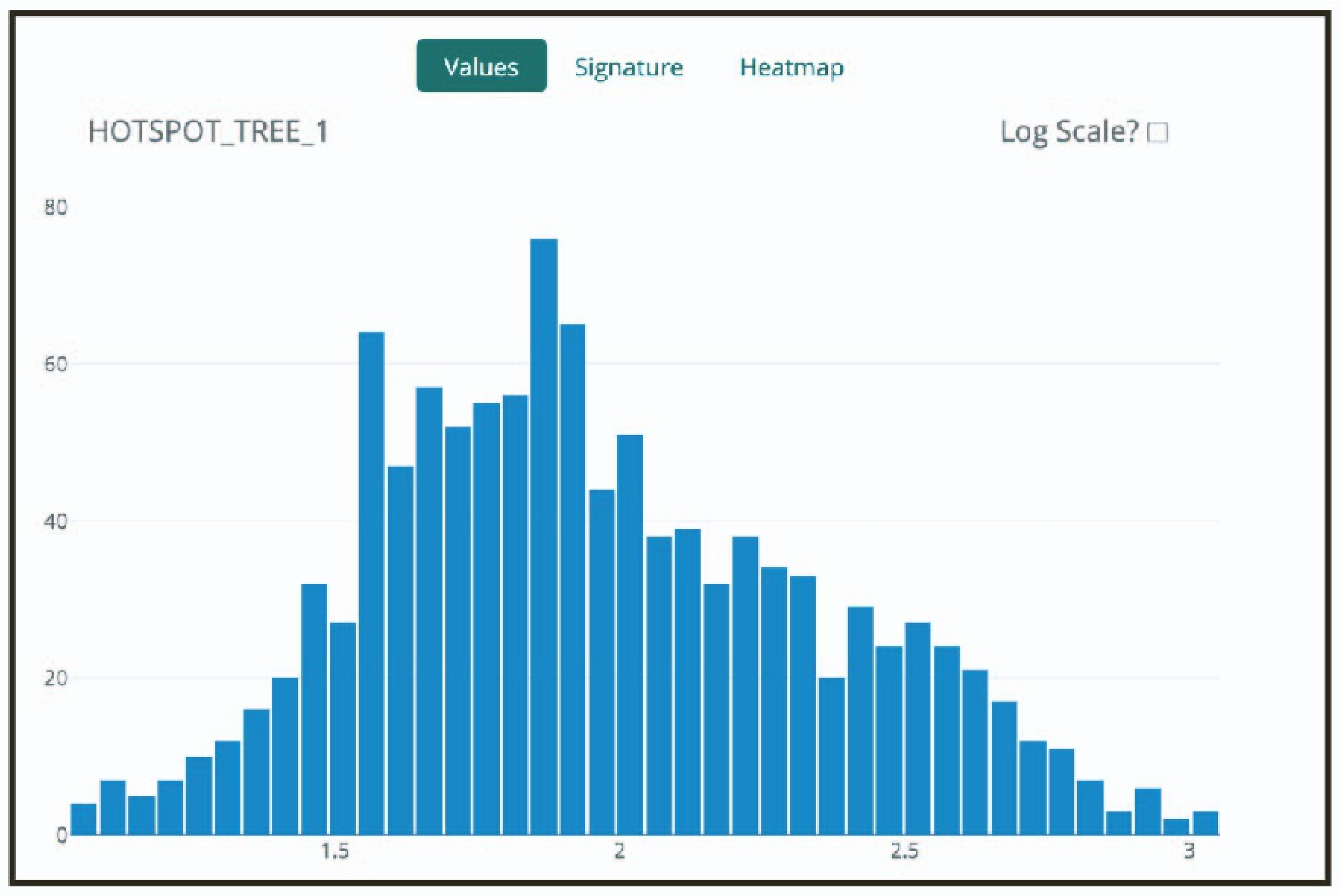

When selecting a Hotspot module, the exploration panel displays the distribution of scores.

Selecting the “log scale” radio button shows the logged distribution.

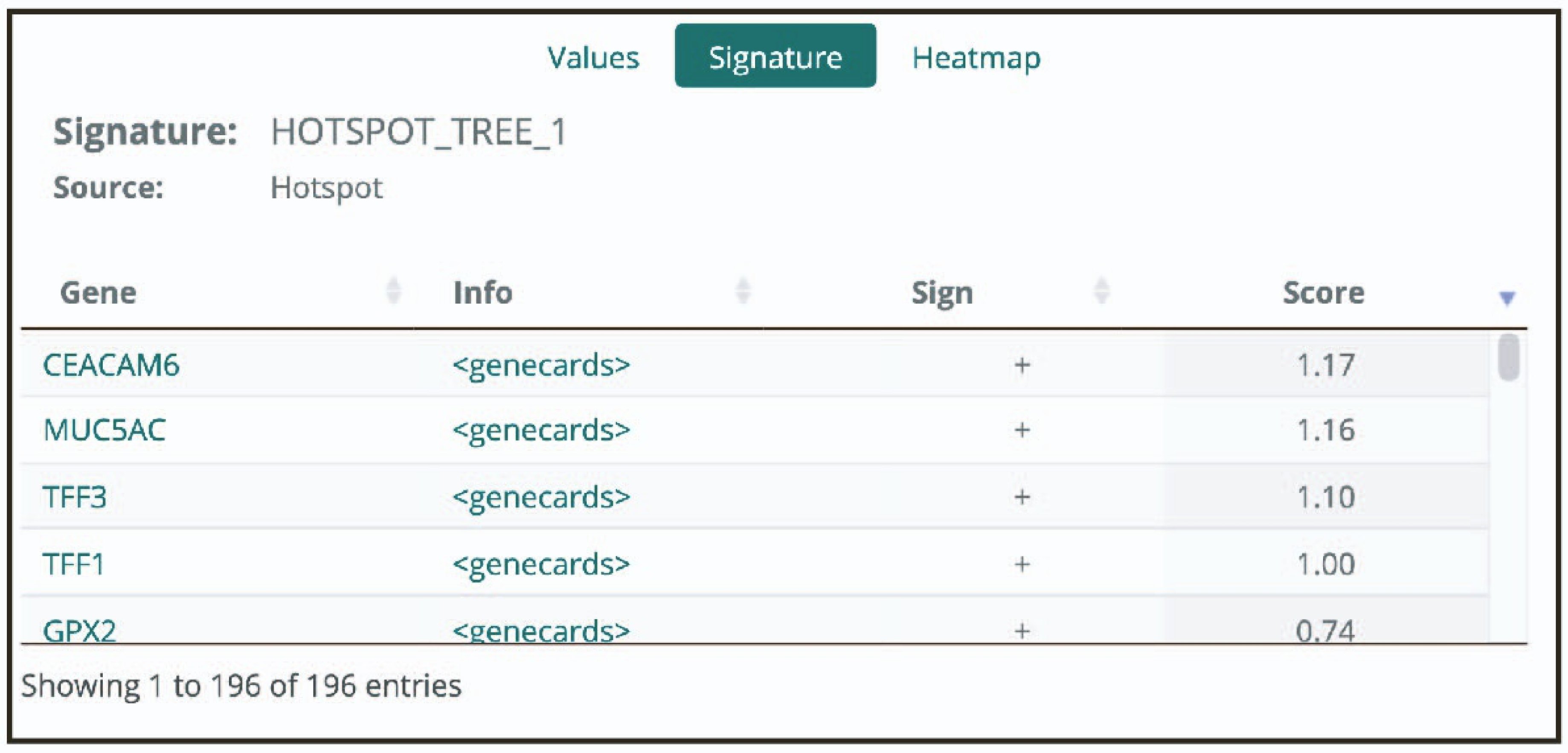

The “Signature” tab shows the genes in the Hotspot gene module.

Clicking on a gene name displays the counts on the cell-projection and tree.

Clicking on “genecards” directs the user to the gene-card entry for the gene of interest

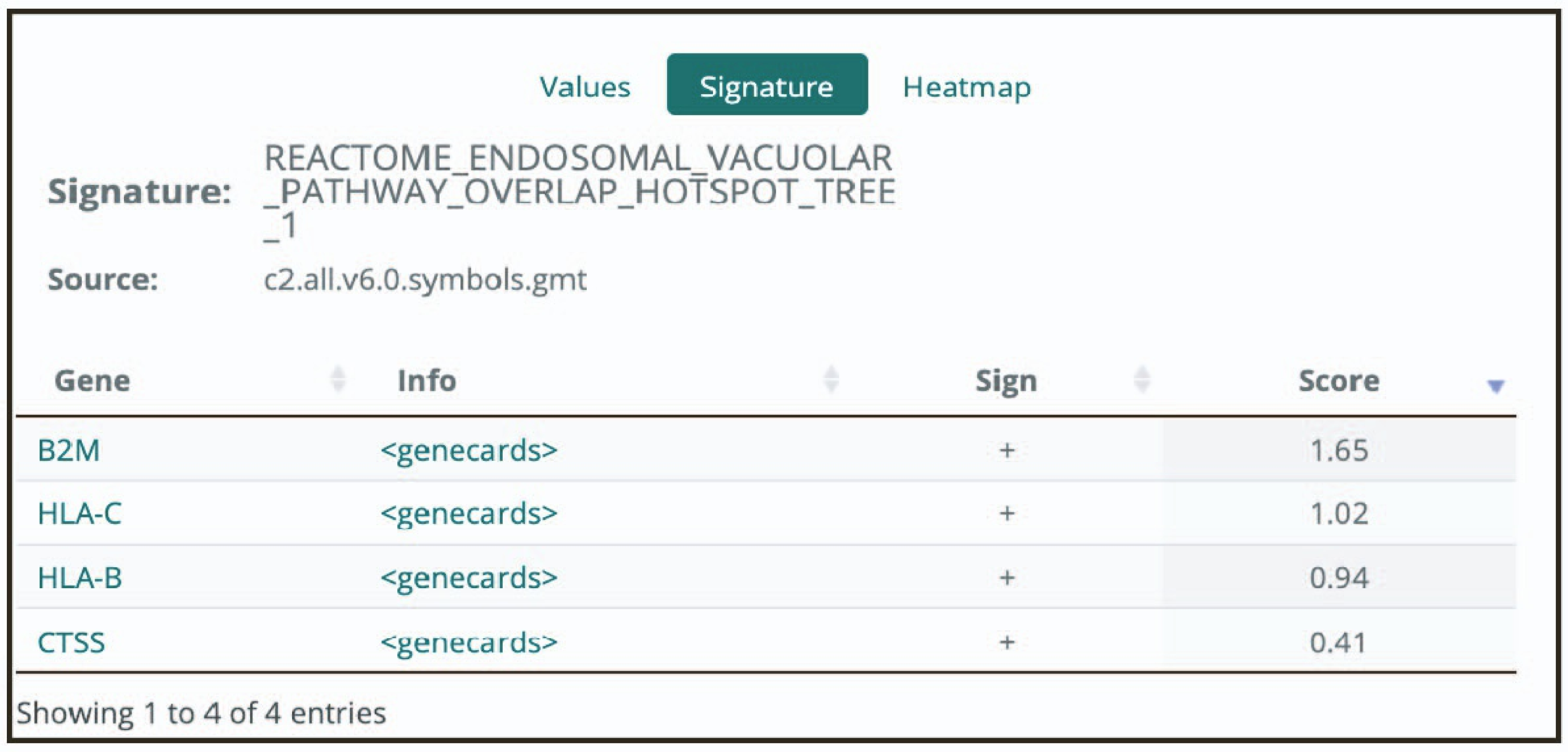

If a signature is selected in the Hotspot mode, the overlapping genes are dispalyed in the “Signature” information tab.

Figure S3: **Elements of the *Hotspot* mode signature exploration panel, related to Figure 1.** Users can view the distribution of scores in the *Hotspot* mode on both normal and log scales. If the *Hotspot* module is selected, scores are the *VISION*-computed score for the genes in the *Hotspot* module. If a signature is selected, the distribution displayed is the signature score of only the genes overlapping between the *Hotspot* module and the gene signature. Users can also view the genes in a *Hotspot* module, or in the overlap between a gene signature and *Hotspot* module.

Display Legend

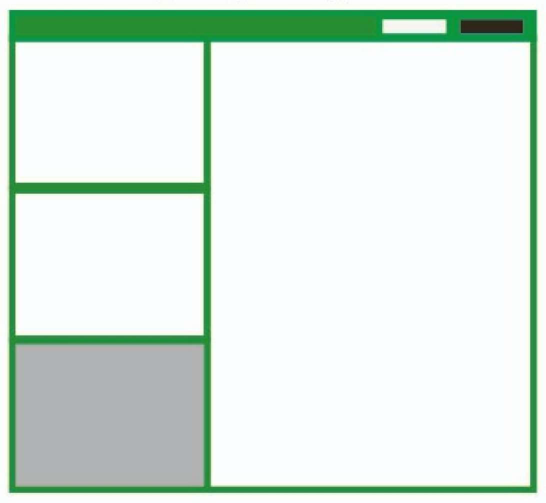

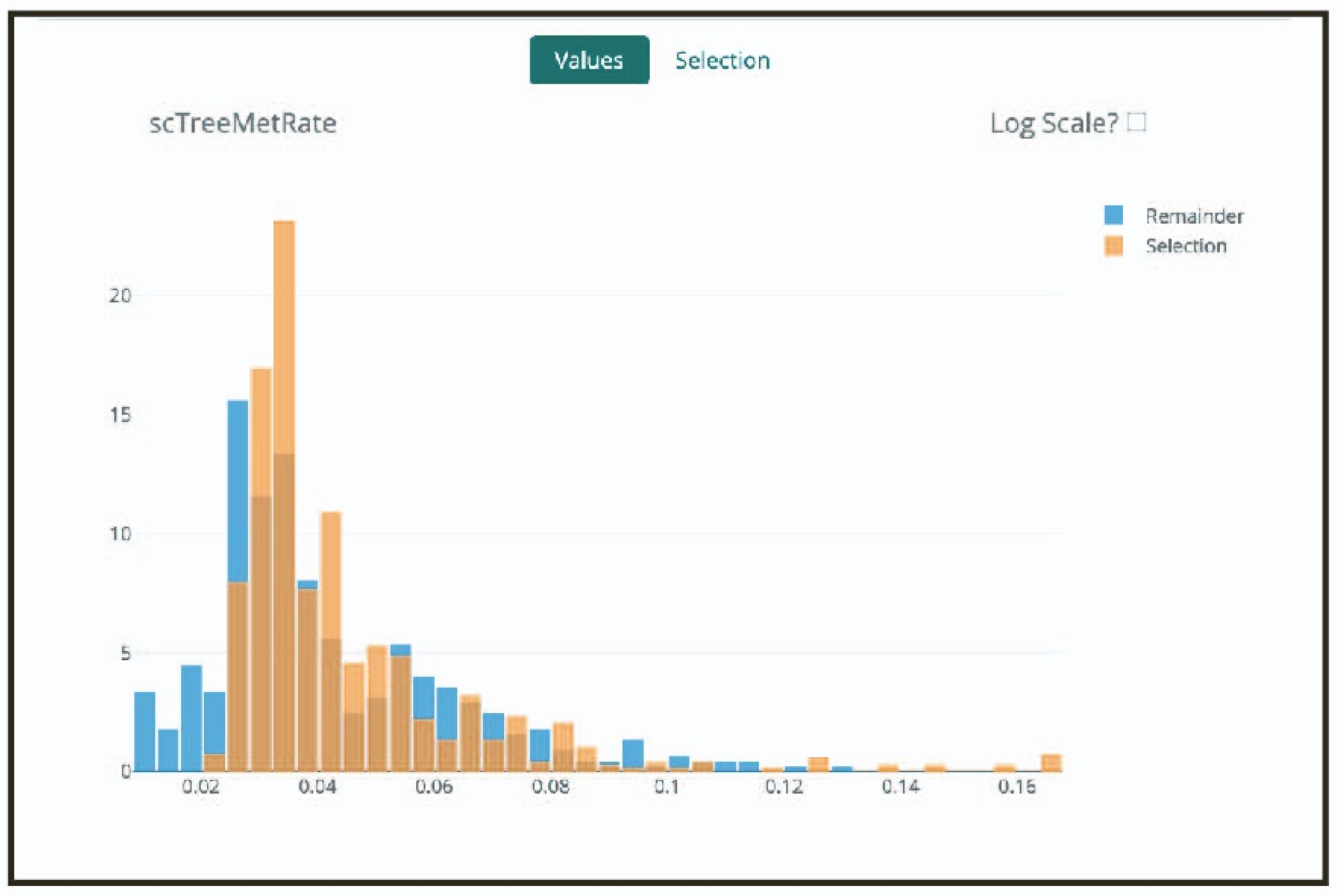

If a cell selection is active, the “Values” distribution will show two distributions: one for the selected cells (orange) and one for the rest of the cells (blue).

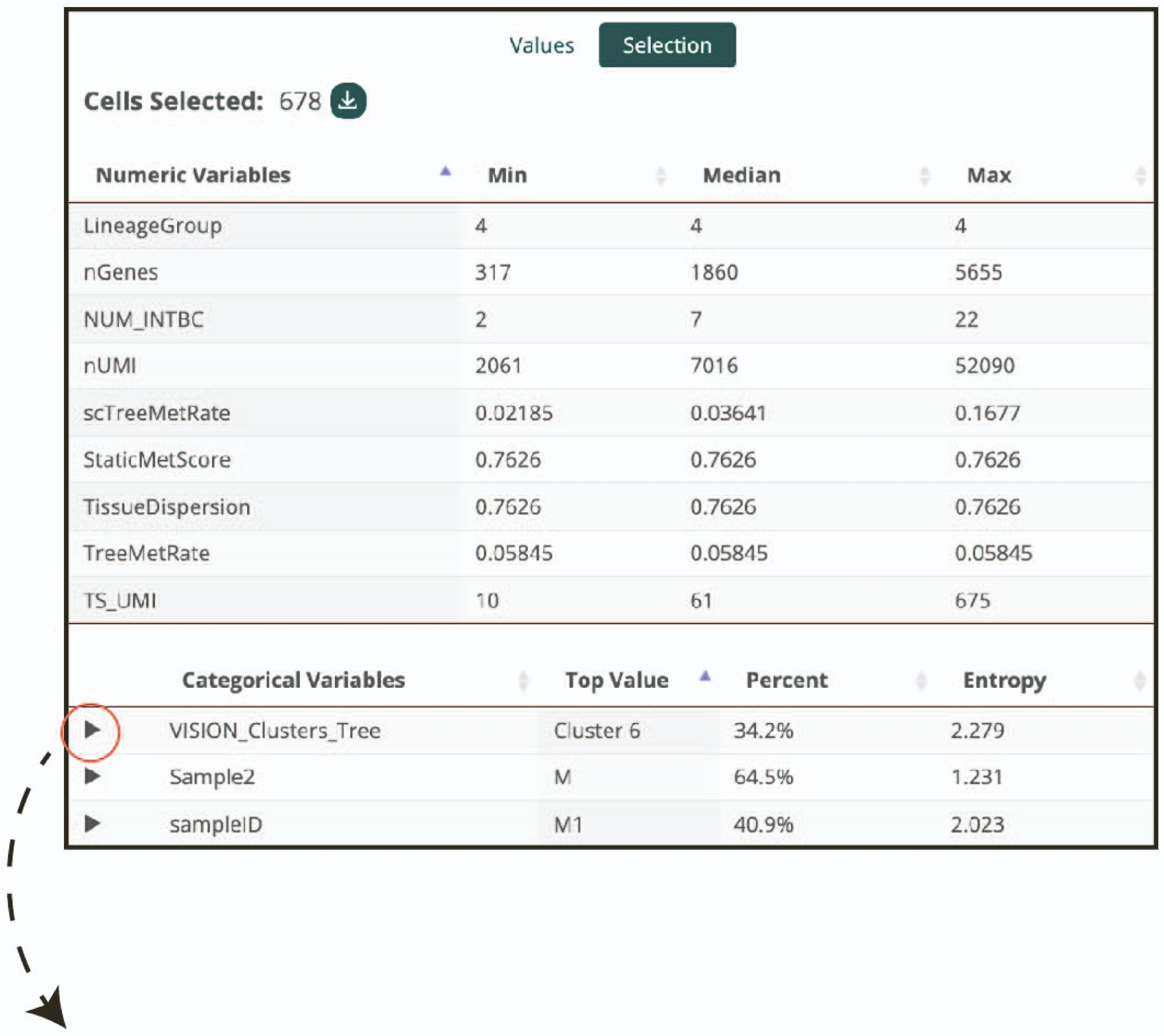

Values | Selection

Cells Selected: 678

Numeric Variables	Min	Median	Max
LineageGroup	4	4	4
nGenes	317	1860	5655
NUM_INTBC	2	7	22
nUMI	2061	7016	52090
scTreeMetRate	0.02185	0.03641	0.1677
StaticMetScore	0.7626	0.7626	0.7626
TissueDispersion	0.7626	0.7626	0.7626
TreeMetRate	0.05845	0.05845	0.05845
TS_UMI	10	61	675

Categorical Variables	Top Value	Percent	Entropy
VISION_Clusters_Tree	Cluster 6	34.2%	2.279
Sample2	M	64.5%	1.231
sampleID	M1	40.9%	2.023

The “Selection” tab will show a breakdown of meta data within the selected cell population.

Summary statistics are shown for numerical meta data.

The most frequent category, percentage of cells reporting this category, and entropy of the variable are shown for categorical meta data.

A list of selected calls can be downloaded with the arrow button next to “Cells Selected”.

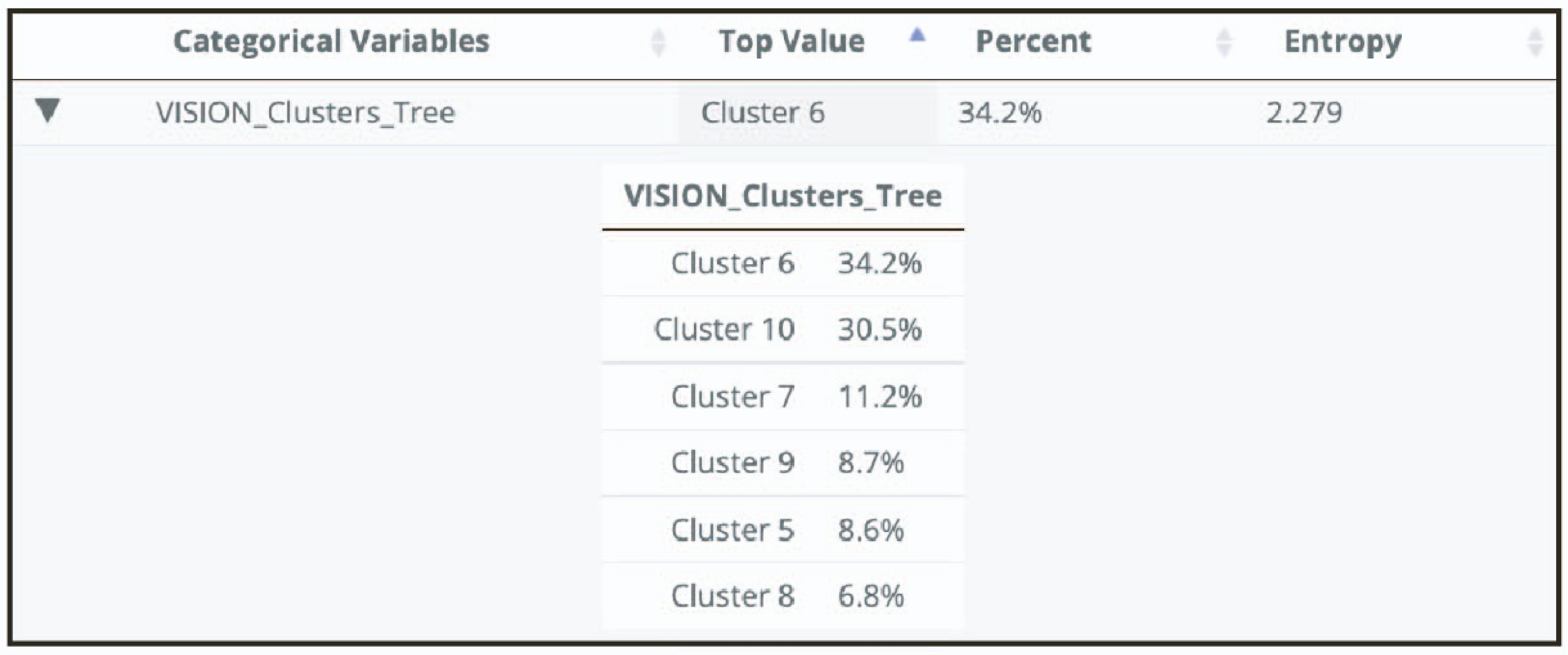

Categorical Variables	Top Value	Percent	Entropy
VISION_Clusters_Tree	Cluster 6	34.2%	2.279

VISION_Clusters_Tree	
Cluster 6	34.2%
Cluster 10	30.5%
Cluster 7	11.2%
Cluster 9	8.7%
Cluster 5	8.6%
Cluster 8	6.8%

Clicking on an arrow next to a categorical variable reveals a more granular breakdown of the composition of a given selection of cells.

Figure S4: **Investigating cell selections, related to Figure 1.** Users can investigate a selection with respect to its distribution of the plotted value as compared to the background (i.e., non-selected cells). *PhyloVision*'s UI also provides a breakdown of metadata within a given selection, including summary statistics over numerical metadata as well as measures of entropy and composition of categorical metadata.
